# The heterobivalent (SSTR2/albumin) radioligand [^67^Cu]Cu-NODAGA-cLAB4-TATE enables efficient somatostatin receptor radionuclide theranostics

**DOI:** 10.7150/thno.100091

**Published:** 2024-08-26

**Authors:** Martin Ullrich, Robert Wodtke, Florian Brandt, Robert Freudenberg, Jörg Kotzerke, Susan Richter, Klaus Kopka, Jens Pietzsch

**Affiliations:** 1 Helmholtz-Zentrum Dresden-Rossendorf, Institute of Radiopharmaceutical Cancer Research, Dresden, Germany.; 2 University Hospital Carl Gustav Carus at the Technische Universität Dresden, Klinik und Poliklinik für Nuklearmedizin, Dresden, Germany.; 3 University Hospital Carl Gustav Carus at the Technische Universität Dresden, Institute of Clinical Chemistry and Laboratory Medicine, Dresden, Germany.; 4 Technische Universität Dresden, School of Science, Faculty of Chemistry and Food Chemistry, Dresden, Germany.; 5 German Cancer Consortium (DKTK), Partner Site Dresden, Dresden, Germany.; 6 National Center for Tumor Diseases (NCT), Partner Site Dresden, University Cancer Center (UCC), Dresden, Germany.

**Keywords:** albumin binder, SPECT imaging, dosimetry, copper-67, pheochromocytoma, allograft model

## Abstract

Somatostatin type 2 receptor (SSTR2) radionuclide therapy using β^-^ particle-emitting radioligands has entered clinical practice for the treatment of neuroendocrine neoplasms (NENs). Despite the initial success of [^177^Lu]Lu‑DOTA-TATE, theranostic SSTR2 radioligands require improved pharmacokinetics and enhanced compatibility with alternative radionuclides. Consequently, this study evaluates the pharmacokinetic effects of the albumin-binding domain cLAB4 on theranostic performance of copper‑67-labeled NODAGA-TATE variants in an SSTR2-positive mouse pheochromocytoma (MPC) model.

**Methods:** Binding, uptake, and release of radioligands as well as growth-inhibiting effects were characterized in cells grown as monolayers and spheroids. Tissue pharmacokinetics, absorbed tumor doses, and projected human organ doses were determined from quantitative SPECT imaging in a subcutaneous tumor allograft mouse model. Treatment effects on tumor growth, leukocyte numbers, and renal albumin excretion were assessed.

**Results:** Both copper‑64- and copper‑67-labeled versions of NODAGA-TATE and NODAGA-cLAB4‑TATE showed similar SSTR2 binding affinity, but faster release from tumor cells compared to the clinical reference [^177^Lu]Lu‑DOTA-TATE. The bifunctional SSTR2/albumin-binding radioligand [^67^Cu]Cu‑NODAGA-cLAB4‑TATE showed both an improved uptake and prolonged residence time in tumors resulting in equivalent treatment efficacy to [^177^Lu]Lu‑DOTA-TATE. Absorbed doses were well tolerated in terms of leukocyte counts and kidney function.

**Conclusion:** This preclinical study demonstrates therapeutic efficacy of [^67^Cu]Cu‑NODAGA-cLAB4‑TATE in SSTR2-positive tumors. As an intrinsic radionuclide theranostic agent, the radioligand provides stable radiocopper complexes and high sensitivity in SPECT imaging for prospective determination and monitoring of therapeutic doses* in vivo*. Beyond that, copper‑64- and copper‑61-labeled versions offer possibilities for pre- and post-therapeutic PET. Therefore, NODAGA-cLAB4-TATE has the potential to advance clinical use of radiocopper in SSTR2-targeted cancer theranostics.

## Introduction

Peptide receptor radionuclide therapy (PRRT) using radiolabeled somatostatin analogs is in clinical use for the treatment of neuroendocrine neoplasms (NENs) for many years, in particular for metastatic or unresectable progressive cases with a high tissue concentration of somatostatin type 2 receptors (SSTR2) 1. This also includes metastatic or inoperable pheochromocytomas and paragangliomas (PCC/PGL) arising from catecholamine-producing chromaffin cells of the adrenal gland or extra-adrenal ganglia, respectively 2. Most PCC/PGL exhibit high SSTR2 levels, and several clinical studies suggest that PRRT is one of the most effective therapies for metastatic cases 2-5.

At present, **[^177^Lu]Lu‑DOTA-TATE** is the most frequently used radiolabeled somatostatin analog in patients with SSTR2-positive NENs owing to its high receptor affinity and selectivity, favorable efficacy, and minimal adverse effects 1, 6-8. Chelate-bound lutetium‑177 undergoes β^-^ decay with a physical half-life of 6.65 days, emitting β^-^ particles and conversion electrons with a mean kinetic energy of 147 keV and a maximum energy of 497 keV, corresponding to ranges of 0.28 and 1.8 mm in soft tissue, respectively 9, 10. To date, **[^177^Lu]Lu‑DOTA-TATE** is approved for treatment of metastatic or unresectable progressive gastroenteropancreatic NENs in many countries, based on results from the NETTER‑1 phase III trial 11. An ongoing phase II trial (NCT03206060) is currently evaluating **[^177^Lu]Lu‑DOTA-TATE** in metastatic and inoperable PCC/PGL. Nevertheless, many tumors progress eventually after initial response to **[^177^Lu]Lu‑DOTA-TATE,** emphasizing the need to further improve SSTR2-targeted radionuclide therapies 12, 13.

Besides its recent success, **[^177^Lu]Lu‑DOTA-TATE** therapy is facing several challenges: First, the short biological half-life of **[^177^Lu]Lu‑DOTA-TATE** in blood limits the uptake in tumors 14, 15. So far, improvements in blood retention and tumor uptake have been reported using the albumin-binding variant **[^177^Lu]Lu‑DOTA‑EB-TATE**, which is currently evaluated in clinical phase I and II trials (NCT05475210, NCT03478358) 15, 16. Second, worldwide increase in lutetium‑177-based endoradiotherapies has raised concerns whether the production capacities of clinical grade lutetium‑177 via nuclear reactors are sufficient to meet the growing demand 17, 18. Third, the relatively long physical half-life of lutetium‑177 is challenging for patient care and, from a radiobiologic standpoint, may not provide the most effective dose rate 19, 20. Fourth, the low relative abundance of γ photons from lutetium‑177 decay (208 keV [11.1 %], 113 keV [6.6 %]) is suboptimal for SPECT imaging 21. Therefore, **[^68^Ga]Ga‑DOTA-TATE** is used as a diagnostic match for prospective determination of therapeutic doses based on PET imaging 22. However, gallium‑68 has a much shorter physical half-life, and concerns have been raised regarding the accuracy of this approach 23. Therefore, concepts directed at improving both pharmacokinetic properties and options for theranostic radionuclide pairs have entered the design of SSTR2-targeted radiopharmaceuticals 24-26.

Copper is an element that provides both therapeutic and diagnostic radionuclides (β^-^ therapy: copper‑67; PET imaging: copper‑64, copper‑61) and thus true theranostic radionuclide pairs 27. Beyond that, copper‑67 offers nuclear properties to be used as a theranostic single radionuclide. Since copper‑67 undergoes β^-^ decay with a half-life of 2.6 days, emitting β^-^ particles with a mean energy of 150 keV 9, it is expected to provide similar therapeutic efficacy as lutetium-177. Higher relative abundance of γ photons from decay of copper‑67 (93 keV [16 %], 185 keV [49 %] compared to lutetium-177 promises higher sensitivity in SPECT imaging 21, 27. Furthermore, copper‑67 can be produced independently of nuclear reactors using particle accelerator methods 28. Advances in copper‑67 production via photonuclear reaction and commercial availability in sufficient quantities and purity have facilitated its potential use in SSTR2-targeted radionuclide therapies 27. Theranostic application of both [^64^Cu]Cu‑SARTATE and [^67^Cu]Cu‑SARTATE for management of SSTR2-positive NENs is currently evaluated in several phase I and IIa trials (NCT04023331, NCT04438304, NCT04440956, NCT03936426).

**Figure 1:**
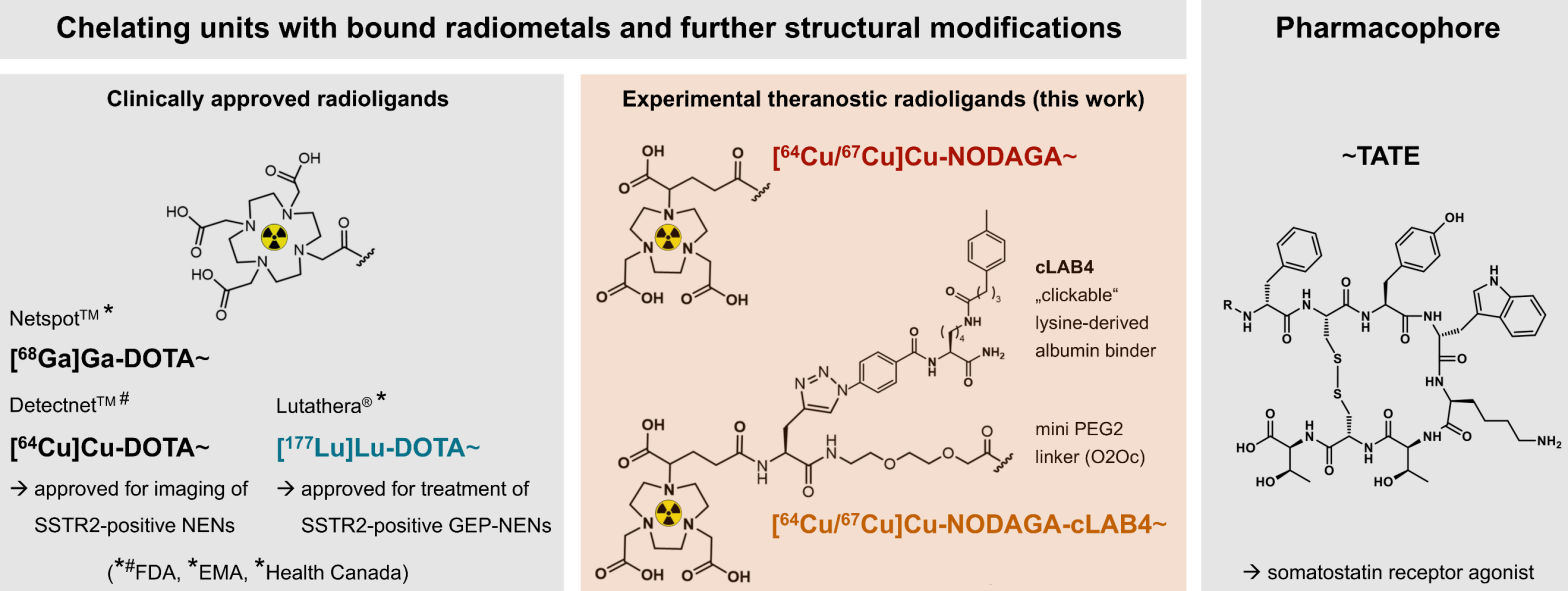
Clinically approved TATE variants for theranostic application in the management of SSTR2-positive neuroendocrine neoplasms and experimental variants (this work) for theranostic use of radiocopper; pharmacokinetic properties of the diagnostic variants **[^64^Cu]Cu‑NODAGA-TATE** and **[^64^Cu]Cu‑NODAGA‑cLAB4-TATE** in tumor-bearing mice have been reported previously 24; (cLAB4) 'clickable' lysine-derived albumin binder 4; (EMA) European Medicines Agency; (FDA) Food and Drug Administration of the United States; (GEP) gastroenteropancreatic; (NENs) neuroendocrine neoplasms.

Towards improvements in both pharmacokinetic properties and radionuclide options, a series of TATE variants equipped with the chelator NODAGA and different 'clickable' lysine-derived albumin binders (cLABs) has been developed in our group (Figure 1) 24. As a commercially available building block, NODAGA provides copper complexes with favorable stability, i.e. kinetic inertness, *in vivo* compared to DOTA 29. Investigations in SSTR2-positive pheochromocytoma allograft mice demonstrated that the albumin-binding **[^64^Cu]Cu‑NODAGA-cLAB4‑TATE** has favorable pharmacokinetic properties resulting in increased uptake and retention in tumors compared to its non-albumin-binding counterpart. Therefore, preclinical testing of the radiotherapeutic variant **[^67^Cu]Cu‑NODAGA-cLAB4‑TATE** is warranted.

The objective of this work is to evaluate the theranostic performance of β^-^ particle-emitting **[^67^Cu]Cu‑NODAGA-cLAB4‑TATE** and **[^67^Cu]Cu‑NODAGA-TATE** in comparison to **[^177^Lu]Lu‑DOTA-TATE** in a mouse pheochromocytoma (MPC) model representing a subtype of SSTR2-positive NENs with a natural receptor biology. This preclinical study reports on (i) cellular binding constants, uptake, and release of the radioligands, also including their copper‑64‑labeled counterparts, (ii) radioligand uptake and growth inhibition in tumor spheroids, (iii) pharmacokinetics, radiation doses, and anti-tumor effects measured in a tumor allograft mouse model using quantitative SPECT imaging, and (iv) hematologic and renal effects. Beyond that, effects due to changes in the molar activity of the radioligands are addressed.

## Materials and methods

### Cells, substances, and radionuclides

MPC cells (clone 4/30PRR, passages 35-40) were routinely cultured in collagen-coated flasks as described previously 30. All commercial reagents and solvents were used without further purification. DOTA-TATE was purchased from Bachem (Bubendorf, Switzerland). **NODAGA-TATE** and **NODAGA-cLAB4‑TATE** were synthesized and chemically characterized as reported previously 24. The non-radioactive metalated conjugates **[^nat^Cu]Cu‑NODAGA-TATE**, **[^nat^Cu]Cu‑NODAGA-cLAB4‑TATE** and **[^nat^Lu]Lu‑DOTA-TATE** were prepared under the same conditions as applied for radiolabeling (see below) using CuSO_4_ (1.2 eq.) and LuCl_3_ (1.2 eq.). After RP-HPLC purification, the compounds were obtained in a purity >95 % (230 nm). [^68^Ga]GaCl_3_ was obtained from the commercially available radionuclide generator IGG 100-50M (Eckert und Ziegler, Berlin, Germany). [^177^Lu]LuCl_3_ was purchased from ITM Isotope Technologies Munich (Garching bei München, Germany). [^67^Cu]CuCl_2_ was purchased from Iotron Medical (Columbia City, IN, USA) in cooperation with CIIC Canadian Isotope Innovations Corp. (Saskatoon, SK, Canada). [^64^Cu]CuCl_2_ was produced at the Helmholtz-Zentrum Dresden-Rossendorf as reported previously 31, 32.

### Radiolabeling of SSTR2 ligands

Radiolabeling of SSTR2 ligands with gallium‑68 and copper‑64 was performed in ammonium acetate buffer (2.0 mol/L, pH 4.5 and pH 5.3, respectively) at 80 °C for 20 min. Radiolabeling with lutetium‑177 and copper‑67 was performed in sodium ascorbate buffer (0.15 mol/L, pH 4.8) for 40 min, at 80 °C and 40 °C, respectively. Radioligand preparations were tested for radiochemical purities > 96 % using analytical radio-high performance liquid chromatography (radio-HPLC) as described previously 33. Molar activities (*A*_m_) of the preparations refer to the activity of radioligand per mole of metal-free ligand and were adjusted to 20, 25, and 40 MBq/nmol as indicated for specific experiments.

### Binding of radioligands to intact cells

Binding of SSTR2 radioligands to intact MPC cells and cell homogenates was measured and analyzed as previously described 34. In brief, all radioligands were prepared at a molar activity of 25 MBq/nmol. Total binding was measured in a series of increasing radioligand concentrations between 0.3 and 40 nmol/L. Activities within in the dilution series were measured and the actual transferred radioligand concentrations were corrected accordingly. Non-specific binding was measured in presence of 1 µmol/L acetyl-TATE. Protein content of cell homogenates was measured at *A*_280 nm_ using a nanodrop spectrophotometer (Thermo Fisher Scientific, Waltham, MA, USA). Dissociation constants (*K*_d_) and maximum binding capacities (*B*_max_) were calculated from regression analysis using the non-linear 'one‑site total, accounting for ligand depletion' and the 'linear non‑specific' binding models implemented in Prism 10 (GraphPad Software, San Diego, CA, USA). Binding experiments with MPC cell homogenates were performed as described elsewhere 24.

### Uptake of radioligands in cells

Uptake of SSTR2 radioligands was measured and analyzed as previously described, with some modifications 34. In brief, radioligands were prepared at a molar activity of 25 MBq/nmol. Total binding and uptake were measured at a final concentration of 20 nmol/L. Non-specific binding and uptake were measured in presence of 1 µmol/L acetyl-TATE. The uptake fraction of radioligands was measured after acid wash with Dulbecco's phosphate-buffered saline containing 0.9 mmol/L CaCl_2_, 0.5 mmol/L MgCl_2_, and 0.05 mol/L glycine, pH 2.8, for 5 min.

### Release of radioligands from cells

MPC cells (2.25 × 10^5^/well) were seeded into collagen coated CELLSTAR 48‑well microplates (Greiner Bio‑One, 67180) and grown for three days. Cell culture medium and washing buffer were pre-warmed at 37 °C, unless specified otherwise. Radioligands were prepared at a molar activity of 40 MBq/nmol. Cells were incubated with the radioligands at a final concentration of 2.5 nmol/L in 0.5 mL of cell culture medium for 1 hour at 37 °C. Non-specific binding was determined in presence of 1 µmol/L acetyl-TATE. Binding to plastic surfaces was determined in cell-free cavities. At the end of radioligand exposure, incubation medium was removed, and cells were washed three times with Dulbecco's phosphate-buffered saline containing 0.9 mmol/L CaCl_2_ and 0.5 mmol/L MgCl_2_. Cells were overlayed with 0.5 mL of radioligand-free medium and further incubated at 37 °C for a total duration of 24 hours. At specific sampling points (1, 2, 5, and 24 hours), supernatants were removed from all cavities. Cell sample cavities were washed with ice-cold wash buffer and lysed with 0.1 mol/L NaOH(aq) containing 1 % (w/v) of sodium dodecyl sulfate. Remaining cavities were re-filled with radioligand-free medium and incubated until the next sampling point. Activity of cell homogenates was measured using the gamma counter Wizard^2^ 2480 (PerkinElmer, Waltham, MA, USA). Release kinetics were analyzed by non-linear regression using the 'two phase decay' model implemented in Prism 10 (GraphPad Software) with the 'Top' and 'Bottom' parameters constrained at 100 %, and 0 %, respectively.

### General animal experimentation

All animal experiments were carried out according to the guidelines of the German Regulations for Animal Welfare and have been approved by the local Ethical Committee for Animal Experiments. MPC cells (4 × 10^6^) were injected subcutaneously into the shoulder of 10-14‑week‑old female nude mice (Rj:NMRI-*Foxn1^nu/nu^*, Janvier Labs, Le Genest-Saint-Isle, France). Prior to treatment and imaging procedures, anesthesia was induced and maintained with inhalation of 10 % (v/v) desflurane in 30/10 % (v/v) oxygen/air. During anesthesia, animals were continuously warmed at 37 °C. Body weight was monitored three times per week and tumor diameters were measured using a caliper. Tumor volume was calculated assuming a tri‑axial ellipsoid with the diameters *a*, *b*, and *c* using the formula *V* = *π*/6 × *a* ×* b* ×* c*. For urine collection, animals were separated in clear conventional rodent boxes. After spontaneous micturition, voided urine was aspirated with a pipette tip, diluted 1:2 with ultrapure water and stored at -80 °C until further processing. For blood sampling, animals were placed in a restrainer and 50 µL of blood were collected from a lateral tail vein using a syringe needle rinsed with heparin. Blood samples were mixed with 4 µL of a 10 % (w/v) aqueous potassium-EDTA solution and kept at 4 °C until further processing. Animals were sacrificed using CO_2_ inhalation and cervical dislocation.

### Treatment and SPECT imaging of mice

Tumor-bearing mice were treated with the SSTR2 radioligands at a single initial activity dose of 50 MBq delivered in 0.2 mL of 0.154 mol/L NaCl(aq) through injection into a tail vein (see *Supporting information,* Table S 1 for further details). Small-animal single-photon emission computed tomography (SPECT) was performed using the Mediso NanoScan® SPECT/CT (Mediso Medical Imaging Systems, Budapest, Hungary). Images were acquired within the following time windows after radioligand injection (with corresponding scan times): 0.75-1 h (30 min), 5-6 h (45 min), 19-26 h (60 min), 40-52 h (60 min), 62-75 h (60 min), and 4-7 d (60 min). With each SPECT scan, a corresponding CT image was recorded and used for anatomical referencing and attenuation correction. Details on image acquisition and reconstruction parameters are included in the *Supporting information*.

### Quantitative analysis of SPECT images and dose calculations in mice

Images were post-processed and analyzed using ROVER version 3.0.77h (ABX GmbH, Radeberg, Germany) and displayed as maximum intensity projections at indicated scaling without decay correction. Regions of interest (ROIs) were generated using tissue-specific signal intensity thresholds. ROI-averaged activity concentrations in tissues [MBq/mL] were determined. Details on SPECT image resolution, image quantification tests, and tissue delineation are included in the *Supporting information.*

Unless specified otherwise, all activity values are reported without decay correction. Time courses of normalized activity concentrations [% initial *A*/mL] (organs, tumors) or normalized activity amounts [% initial *A*] (total body, urinary bladder) were analyzed by non-linear regression using the 'One-phase decay', 'Plateau followed by one-phase decay', or 'Two-phase decay' models implemented in Prism 10 (GraphPad Software), with extrapolated *y*‑values approaching a lower limit constrained to 0. The normalized time-cumulated activity per milliliter of tissue [% initial *A*/mL × d] was calculated from areas under time-activity curves. For dose estimation in tumors and kidneys, these values were further converted into total time-cumulated organ activity per initial activity dose [MBq × h/MBq] using a realistic dosimetry model for 30‑g‑mice 35 implemented in Olinda 2.2.3 (Hermes Medical Solutions AB, Stockholm, Sweden). The physical density of tissues was assumed to be 1 g/cm^3^. The radionuclide-specific mean absorbed tissue dose *D*_T_ [Gy] in tumors and kidneys was calculated using the sphere model in Olinda 2.2.3 (Hermes Medical Solutions AB).

### Projected human dose calculations

Tissue-specific pharmacokinetic profiles of radioligands in mice were transformed into human time scale and organ masses using allometric scaling, and dose calculations were performed as reported elsewhere 36, with some modifications. In brief, projected cumulated activity in human organs was calculated by curve fitting as described above for investigations in mice. Calculations were performed separately for female and male subjects. Cumulated activity in the urinary bladder was determined assuming a voiding interval of four hours. Projected human doses were calculated for male and female subjects using the ICRP 89 Adult Female (60 kg) and ICRP 89 Adult Male (73 kg) models implemented in Olinda 2.2.3 (Hermes Medical Solutions AB).

### Leukocyte counting in blood samples

Blood samples were diluted 1:5 with 0.154 mol/L NaCl(aq) containing 3 % (v/v) acetic acid and incubated for 15 min at room temperature to ensure complete disruption of red blood cells. Leukocyte numbers were determined microscopically using a Neubauer hemocytometer and reported as cell concentration present in the original sample [leukocytes/mL].

### Albumin-to-creatinine-ratios in urine samples

Albumin and creatinine concentrations in urine samples were measured on a Cobas c701 module (Roche Diagnostics, Mannheim, Germany) using the ALBT2 Tina-quant Albumin Gen.2 immunologic turbidity test and the CREJ2 Creatinine Jaffé Gen.2 colorimetric test according to the manufacturer's instructions. Data were expressed as urinary albumin-to-creatinine ratio (ACR, [mg/g]).

### Statistical analysis

Statistical analysis was performed using Prism 10 (GraphPad Software). Unless stated otherwise, data are presented as means with standard error and *n* represents the number of experiments. Significance of differences was tested using ANOVA with Šídak *post-hoc* test. Significance of relationships was tested using Pearsons's correlation test. Differences and relationships were considered significant at *p‑*values < 0.05.

## Results

### Cellular binding and uptake of copper‑64- and copper‑67-labeled NODAGA-TATE variants

**Figure 2:**
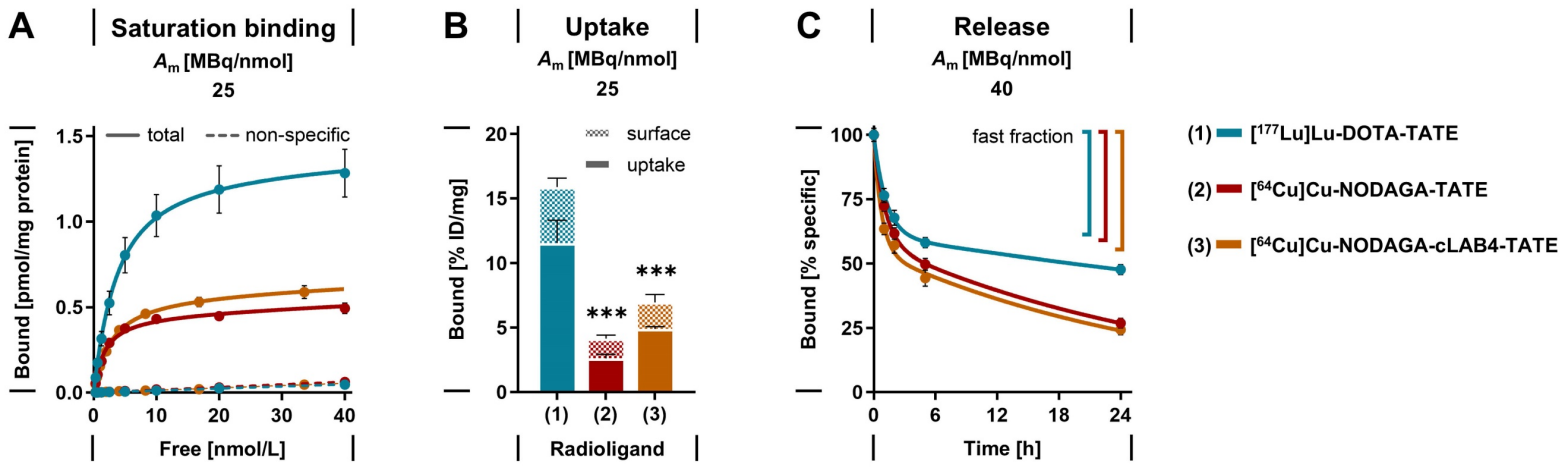
Saturation binding, uptake, and release of lutetium‑177- and copper‑64-labeled TATE variants measured using intact MPC cell monolayers; (A) Total and non-specific binding at radioligand concentrations between 0.3-40 nmol/L (transfer-corrected); (B) Specific binding and effective receptor-mediated uptake at a radioligand concentration of 25 nmol/L; (C) Biphasic release from cells after incubation at a radioligand concentration of 25 nmol/L followed by radioligand removal; cells were incubated with radioligands for 1 hour at 37° C; non-specific binding was determined in presence of 1 µmol/L acetyl-TATE; (*A*_m_) molar activity at incubation start; data presented as means ± standard error; significance of differences compared to **[^177^Lu]Lu‑DOTA-TATE** *** *p* < 0.001.

SSTR2 binding affinities of both copper‑64- and copper‑67‑labeled **NODAGA-TATE** and **NODAGA-cLAB4-TATE** versions were determined to be in the range of clinical references **[^177^Lu]Lu‑DOTA-TATE** and **[^68^Ga]Ga‑DOTA-TATE** with *K*_d_ values between 1.8 and 2.9 nmol/L. **[^64^Cu]Cu‑DOTA-TATE** reached significantly lower *K*_d_ values < 1 nmol/L (Table 1, Figure 2 A-B). Binding capacities of MPC cells for all copper‑64- and copper‑67-labeled TATE variants, regardless of the chelator or albumin-binding domian, were lower compared to the lutetium‑177- and gallium‑68-labeled references, with *B*_max_ values reduced by 32-62 %. Binding constants measured with MPC cell homogenates confirmed these trends (*Supporting information*, Figure S 1).

Competition binding against **[^64^Cu]Cu‑DOTA-TATE** showed similar inhibitory constants (*K*_i_ values between 0.76 and 1.78 nmol/L) for the non-radioactive metalated conjugates **[^nat^Cu]Cu‑NODAGA-TATE**, **[^nat^Cu]Cu‑NODAGA-cLAB4‑TATE**, and **[^nat^Lu]Lu‑DOTA-TATE** as well as for their corresponding metal-free precursors (*Supporting information,* Figure S 2).

Upon SSTR2 binding, all copper‑64- and copper‑67‑labeld TATE variants induced putative endocytosis of the ligand-receptor complex to a similar extent as **[^177^Lu]Lu‑DOTA-TATE** with uptake fractions of 54-72 %. Nevertheless, the effective uptake of **[^64^Cu]Cu‑NODAGA-TATE** and **[^64^Cu]Cu‑NODAGA-cLAB4‑TATE** was 77 % and 58 % lower, respectively, compared to **[^177^Lu]Lu‑DOTA-TATE** (Figure 2 B), which is consistent with the observed trends in *B*_max_ values.

### Cellular release of copper‑64-labeled NODAGA-TATE variants

After initial binding, the release of copper‑64-labeled **NODAGA-TATE** variants from MPC cells was compared to that of **[^177^Lu]Lu‑DOTA-TATE**. All radiolabeled TATE variants showed a biphasic release from the cells in radioligand-free medium (Figure 2 C). During the early phase, similar proportions between 39 and 45 % of the initially bound radioligands were released from the cells as fast fractions with half-lives < 1 hour. During the late phase, the radioligands were released at considerably slower rates, however, the half-lives for the copper‑64-labeled **NODAGA-TATE** variants were significantly shorter compared to **[^177^Lu]Lu‑DOTA-TATE**. The effective release of the copper‑64-labeled **NODAGA-TATE** variants was 22 % higher after 24 hours compared to **[^177^Lu]Lu‑DOTA-TATE**.

The trends in release kinetics and *B*_max_ values observed for the copper‑64-labeled **NODAGA-TATE** are consistent with the uptake and growth-inhibitory effects of the copper‑67-labeled versions measured in MPC tumor spheroids (*Supporting information*, Figure S 3, Figure S 4, Table S 2). Treatment of spheroids with **[^67^Cu]Cu‑NODAGA-TATE** and **[^67^Cu]Cu‑NODAGA-cLAB4‑TATE** required up to six‑fold higher initial activity concentrations in the incubation medium to provide similar efficacy as **[^177^Lu]Lu‑DOTA-TATE**. To explore the potential effects of cLAB4‑mediated albumin binding on tumor uptake and therapeutic efficacy of **[^67^Cu]Cu‑NODAGA-cLAB4‑TATE**, further investigations were performed *in vivo*.

### SPECT imaging of copper‑67-labeled NODAGA-TATE variants in tumor-bearing mice

**Figure 3:**
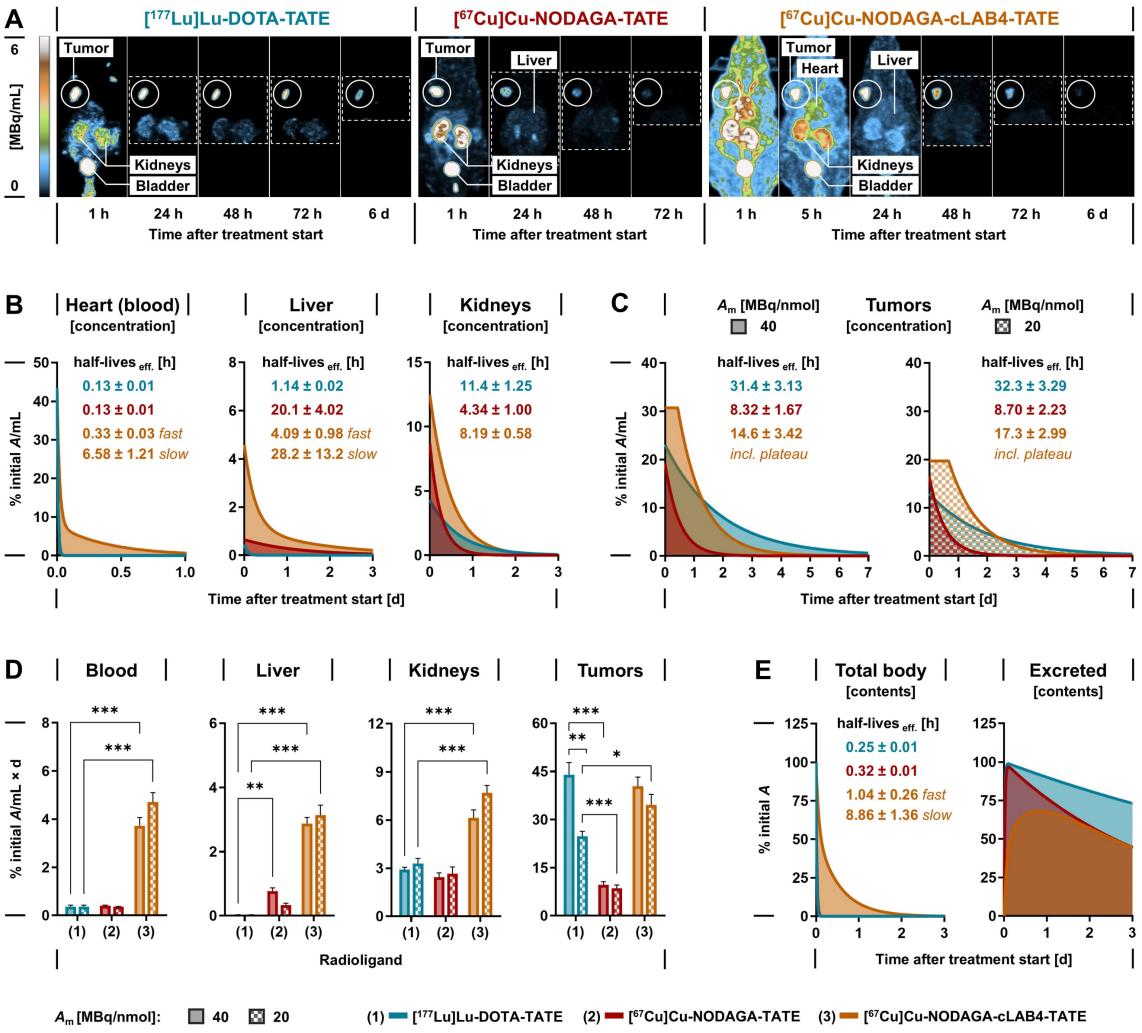
Distribution, tissue-specific pharmacokinetics and cumulated activity of lutetium‑177- and copper‑67-labeled TATE variants in MPC allograft mice measured by quantitative SPECT imaging; animals received 50 MBq of the radioligands each prepared at different molar activities corresponding to molar amounts of 1.25 nmol (*A*_m_ = 40 MBq/nmol) and 2.5 nmol (*A*_m_ = 20 MBq/nmol) at treatment start; (A) Maximum-intensity projections of SPECT images at indicated time points and scaling; image series from treatments with radioligands at a molar activity of 40 MBq/nmol; (B-C) Time-courses of tissue-specific activity concentration after treatment; (D) Tissue-specific mean cumulated volume activity calculated from areas under curves; (E) Time-courses of excreted activity; data presented without decay correction as means ± standard error; significance of differences: * *p* < 0.05 ** *p* < 0.01, *** *p* < 0.001.

The theranostic performance of **[^67^Cu]Cu‑NODAGA-TATE** and **[^67^Cu]Cu‑NODAGA-cLAB4‑TATE** *in vivo* was investigated and compared to **[^177^Lu]Lu‑DOTA-TATE** in subcutaneous MPC allograft mice. After treatment with the therapeutic radioligands at equal initial activity doses, SPECT imaging provided quantitative data on their distribution and tissue kinetics, monitored for up to seven days. All radioligands showed uptake in tumors and kidneys and were excreted via the renal pathway (Figure 3 A). The copper‑67-labeled **NODAGA-TATE** variants provided higher count rates and higher spatial resolution in SPECT imaging compared to **[^177^Lu]Lu‑DOTA-TATE**. Details on image resolution and quantification are included in the *Supporting information* (Figure S 5 A-F).

### Pharmacokinetics of copper‑67-labeled NODAGA-TATE variants in tumor-bearing mice

Quantitative analyses of SPECT images provided time courses of activity concentrations and effective radioligand half-lives in blood, liver, kidneys, and tumors (Figure 3 B-C). Both **[^67^Cu]Cu‑NODAGA-TATE** and **[^177^Lu]Lu‑DOTA-TATE** were rapidly cleared from the blood (effective half-life: 0.13 h) resulting in comparably low cumulated activity (Figure 3 D). The albumin affinity of **[^67^Cu]Cu‑NODAGA-cLAB4‑TATE** was associated with slower biphasic blood kinetics (effective half-lives: 0.33 h distribution; 6.58 h clearance) resulting in up to 13-fold higher cumulated activity.

In the liver, activity concentrations of the copper‑67-labeled **NODAGA-TATE** variants remained low. Nevertheless, uptake, retention, and thus cumulated activity were higher compared to **[^177^Lu]Lu‑DOTA-TATE**, resulting from free [^67^Cu]Cu^2+^ (< 4 %) in the radioligand preparations and a higher tissue residence of **[^67^Cu]Cu‑NODAGA-cLAB4‑TATE**.

In kidneys, uptake of the copper‑67-labeled **NODAGA-TATE** variants was two- to three-fold higher compared to **[^177^Lu]Lu‑DOTA-TATE**. However, the activity concentration of **[^67^Cu]Cu‑NODAGA-TATE** decreased with a shorter effective half-life compared to **[^177^Lu]Lu‑DOTA-TATE** eventually resulting in a similar cumulated activity for both radioligands. The extended blood retention of **[^67^Cu]Cu‑NODAGA-cLAB4‑TATE** delayed the effective clearance from kidneys resulting in a two- to three‑fold higher cumulated activity compared to **[^177^Lu]Lu‑DOTA-TATE**.

In tumors, both **[^67^Cu]Cu‑NODAGA-TATE** and **[^177^Lu]Lu‑DOTA-TATE** showed similar uptake when applied at a molar activity of 40 MBq/nmol. Due to faster release of **[^67^Cu]Cu‑NODAGA-TATE** from tumors (effective half‑life: 8.32 h) 78 % lower cumulated activity compared to **[^177^Lu]Lu‑DOTA-TATE** (effective half-life: 31.4 h) was measured. The albumin-binding variant, **[^67^Cu]Cu‑NODAGA-cLAB4‑TATE,** overcame this shortcoming based on two pharmacokinetic improvements: First, the uptake in tumors increased by 60 %. Second, the initial activity concentration in tumors remained largely stable for up to 14 hours after treatment start resulting in higher tumor retention (effective half‑life: 14.6 h). As a result, the cumulated activity increased four-fold and reached levels similar to **[^177^Lu]Lu‑DOTA-TATE**. Pharmacokinetic profiles resulted in a lower tumor-to-blood ratio of cumulated activities for **[^67^Cu]Cu‑NODAGA-cLAB4‑TATE** (11 ± 1.4) compared to treatments with **[^67^Cu]Cu‑NODAGA-TATE** (24 ± 3.2; *p* < 0.001) and **[^177^Lu]Lu‑DOTA-TATE** (123 ± 19; *p* < 0.001). At the same time, the tumor-to-kidney ratio of cumulated activities was lower in treatment with **[^67^Cu]Cu‑NODAGA-cLAB4‑TATE** (6.7 ± 0.4) compared to **[^177^Lu]Lu‑DOTA-TATE** (15 ± 2.0; *p* < 0.001), but slightly higher compared to **[^67^Cu]Cu‑NODAGA-TATE** (4.3 ± 0.7).

Tumor uptake of all investigated radioligands was attenuated by decreasing their molar activity from 40 to 20 MBq/nmol, with the copper‑67-labeled **NODAGA-TATE** variants being less affected compared to **[^177^Lu]Lu‑DOTA-TATE**. Consequently, this was associated with a 14 % reduction in cumulated activity for the copper‑67-labeled **NODAGA-TATE** variants compared to 44 % for **[^177^Lu]Lu‑DOTA-TATE**.

Both **[^67^Cu]Cu‑NODAGA-TATE** and **[^177^Lu]Lu‑DOTA-TATE** were excreted from the body with similar effective half-lives (0.32 and 0.25 compared to a slower biphasic excretion of **[^67^Cu]Cu‑NODAGA-cLAB4‑TATE**, releasing 55 % of the initial activity with an extended effective half-life during the late phase (8.86 h) (Figure 3 E). Additional SPECT data and details on image-based kinetic analysis are provided in the *Supporting information* (Figures S 6 - S 10, Table S 3).

### Projected human doses for treatment with copper‑67-labeled NODAGA-TATE variants

Projected human pharmacokinetics and radiation doses to organs were estimated based on quantitative SPECT data from female MPC allograft mice treated with 50 MBq of the radioligands at a molar activity of 40 MBq/nmol. Projected time-activity curves, organ doses, total body doses and effective doses showed radioligand-specific results in females and males (*Supporting information,* Figure S 11, Table S 4). As an example, the extrapolated female blood kinetics of **[^67^Cu]Cu‑NODAGA-TATE** were similar to **[^177^Lu]Lu‑DOTA-TATE** (effective half-lives: 0.88 h and 0.83 h, respectively), compared to the slower biphasic kinetics of **[^67^Cu]Cu‑NODAGA-cLAB4‑TATE** (effective half-lives: 2.15 h distribution; 29.0 h clearance).

The organs that received the highest projected human doses were urinary bladder > kidneys > heart wall > liver for **[^67^Cu]Cu‑NODAGA-cLAB4‑TATE**, urinary bladder > kidneys > liver > heart wall for **[^67^Cu]Cu‑NODAGA-TATE**, and urinary bladder > kidneys > heart wall > liver for **[^177^Lu]Lu‑DOTA-TATE**. Projected doses in the red bone marrow were similar for **[^67^Cu]Cu‑NODAGA-TATE** and **[^177^Lu]Lu‑DOTA-TATE**, and up to 17‑fold higher for **[^67^Cu]Cu‑NODAGA-cLAB4‑TATE**. Kidney doses were similar for **[^67^Cu]Cu‑NODAGA-TATE** and **[^177^Lu]Lu‑DOTA-TATE**, and up to 60 % higher for **[^67^Cu]Cu‑NODAGA-cLAB4‑TATE**. The projected total effective dose for treatment with **[^67^Cu]Cu‑NODAGA-TATE** was similar to **[^177^Lu]Lu‑DOTA-TATE**, compared to a 2.3‑fold higher effective dose for **[^67^Cu]Cu‑NODAGA-cLAB4‑TATE**.

### Treatment efficacy of copper‑67-labeled NODAGA-TATE variants in tumor-bearing mice

**Figure 4:**
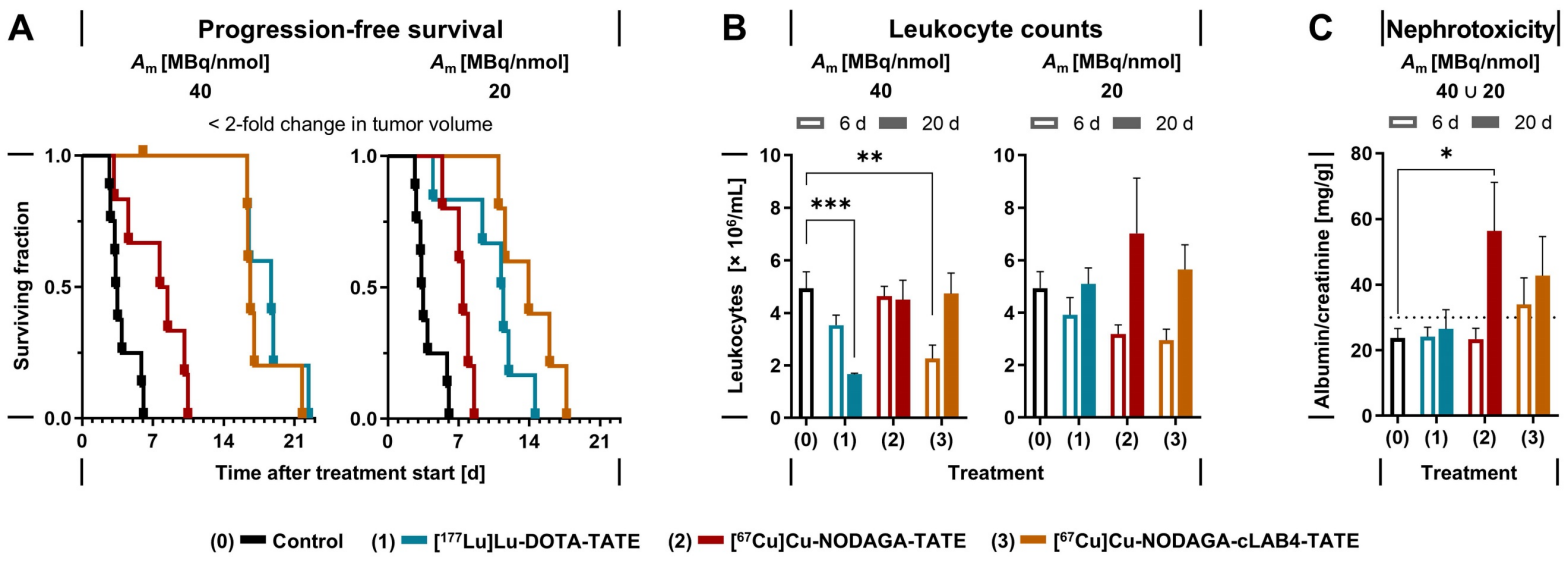
Treatment effects of lutetium‑177- and copper‑67-labeled TATE variants in MPC allograft mice; all animals received an initial activity dose of 50 MBq corresponding to molar amounts of 1.25 nmol (*A*_m_ = 20 MBq/nmol) or 2.5 nmol (*A*_m_ = 20 MBq/nmol) at treatment start; (A) Progression-free survival (PFS) defined as < 2-fold change in tumor volume compared to treatment start; censored data points along horizontal lines indicate animals withdrawn from follow-up before exceeding the PFS threshold; (B) Leukocyte counts in blood at indicated time points after treatment start; (C) Albumin/creatinine ratio (ACR) in urine estimating nephrotoxicity at indicated time points after treatment start, pooled data from treatments at different molar activities; (dotted line) lower threshold for microalbuminuria at ACR > 30 < 300 mg/g; data presented as means ± standard error; significance of differences: * *p* < 0.05, ** *p* < 0.01, *** *p* < 0.001.

Treatments of MPC allograft mice with 50 MBq of copper‑67-labeled **NODAGA‑TATE** variants provided absorbed doses in tumors between 7.82 and 40.2 Gy, resulting in inhibition of tumor growth and thus improvements in progression-free survival of 4 to 13 days compared to vehicle-treated controls. Treatment with **[^67^Cu]Cu‑NODAGA‑cLAB4-TATE** was even more effective compared to **[^67^Cu]Cu‑NODAGA‑TATE** (Figure 4 A).

At a molar activity of 40 MBq/nmol, treatments with **[^67^Cu]Cu‑NODAGA‑cLAB4-TATE** or **[^177^Lu]Lu‑DOTA-TATE** resulted in five-fold longer progression-free survival compared to controls, while **[^67^Cu]Cu‑NODAGA-TATE** provided only a two-fold increase. Reduction of molar activity to 20 MBq/nmol attenuated the responses in progression-free survival by less than 16 % for the copper‑67-labeled **NODAGA-TATE** variants compared to 40 % for **[^177^Lu]Lu‑DOTA-TATE**. Similar trends emerged for overall survival (Table 3). Progression-free and overall survival each showed a significant linear relationship with absorbed doses in tumors. Details on tumor progression and survival analysis are included in the *Supporting information* (Figure S 12).

### Effects of copper‑67-labeled NODAGA-TATE variants on leukocytes and renal function

Treatments of MPC allograft mice with copper‑67-labeled **NODAGA-TATE** variants were tolerated well, without deterioration in general condition or body weight (*Supporting information*, Figure S 13). Moderate decreases in leukocyte numbers and kidney function had no serious implications during follow‑up. Treatments with the copper‑67-labeled **NODAGA-TATE** variants showed less impact on leukocyte numbers compared to **[^177^Lu]Lu‑DOTA-TATE** (Figure 4 B). Six days after treatment start, all treatments were associated with an initial reduction in leukocyte counts between 21 % and 54 %, with slightly stronger decreases when radioligands were applied at molar activities of 40 MBq/nmol. Leukocyte numbers recovered within 20 days after treatment, except for **[^177^Lu]Lu‑DOTA-TATE** applied at a molar activity of 40 MBq/nmol, where leukocyte counts were further reduced by 66 % compared to treatment start.

Elevated albumin/creatinine ratios (ACR) in urine samples provided an estimate on the extent of kidney damage occurring in response to treatments (Table 4). The estimated absorbed doses in kidneys of MPC allograft mice were in the same range for treatments with **[^67^Cu]Cu‑NODAGA-TATE** and **[^177^Lu]Lu‑DOTA-TATE** (2.42-2.81 Gy/50 MBq), compared to a 2.6‑fold higher absorbed dose for **[^67^Cu]Cu‑NODAGA‑cLAB4-TATE** (6.82 Gy/50 MBq). Since changes in molar activity of the radioligands had no significant effects on the absorbed dose in kidneys, ACRs from treatments at 40 and 20 MBq/nmol were analyzed together. The incidence of microalbuminuria (ACR > 30 mg/g) was between 8 % and 33 % higher after treatments with the copper‑67-labeled **NODAGA-TATE** variants compared to **[^177^Lu]Lu‑DOTA-TATE**. Nevertheless, average albumin excretion for all radioligand treatments remained well below the threshold for macroalbuminuria (ACR > 300 mg /g) (Figure 4 C).

## Discussion

This preclinical study, conducted in an SSTR2-positive mouse pheochromocytoma model, demonstrates efficacy of targeted somatostatin receptor radionuclide therapies with **[^67^Cu]Cu‑NODAGA-TATE** and **[^67^Cu]Cu‑NODAGA-cLAB4‑TATE**. Both radioligands provide high SSTR2 affinity, high kinetic inertness of radiocopper complexes in blood, and favorable sensitivity for *in vivo* dosimetry based on quantitative SPECT imaging*.* Although **[^67^Cu]Cu‑NODAGA-TATE** has a relatively short effective half-life in tumors, the heterobivalent SSTR2/albumin-binding compound **[^67^Cu]Cu‑NODAGA-cLAB4‑TATE** showed both an improved initial uptake and prolonged residence time in tumors. Absorbed doses in tumors and survival of tumor-bearing mice demonstrated equivalent therapeutic efficacy of **[^67^Cu]Cu‑NODAGA-cLAB4‑TATE** and **[^177^Lu]Lu‑DOTA-TATE**, with projected human doses in an acceptable range for clinical application. Leukocyte counts and albumin excretion after treatment indicate that **[^67^Cu]Cu‑NODAGA-cLAB4‑TATE** is well tolerated with only mild hematologic and renal adverse effects.

In the present study, SSTR2 radioligands were investigated at molar activities between 20 and 40 MBq/nmol. Herein, the molar activity refers to the ratio between the radiolabeled peptide and its non-radiolabeled (metal-free) counterpart (expressed as activity units per molar units of peptide). A molar activity of 40 MBq/nmol corresponds to recent practice in clinical **[^177^Lu]Lu‑DOTA-TATE** production where molar activities between 40 and 60 MBq/nmol are commonly envisaged 37, 38. On the other hand, a molar activity of 20 MBq/nmol would not meet the criteria for clinical use but was included to investigate the effects of non-radiolabeled peptide contents on the pharmacokinetics of the radioligands.

Preclinical evaluation of theranostic radioligands requires reliable models. As previously reported, the MPC allograft mouse model, in addition to a high natural abundance of SSTR2, also reflects pathophysiological characteristics of human pheochromocytomas, such as increased catecholamine production and hypertension 30, 33, 39.

High target affinities and a high number of binding sites are essential pharmacologic prerequisites for the eligibility of radiolabeled receptor ligands in cancer management. SSTR2 binding affinities in the low nanomolar range measured for both the copper‑64- and copper‑67-labeled versions of **NODAGA-TATE** and **NODAGA-cLAB4‑TATE** support their theranostic potential. This is consistent with the results from previous SSTR2 binding studies using intact MPC cells and homogenates 24, 40. Moreover, a low nanomolar affinity has also been reported for binding of **[^64^Cu]Cu‑NODAGA-TATE** towards membrane preparations of gene-modified human HTC116-SSTR2+ cells (*K*_d_ = 0.5 nmol/L) 41. Beyond that, the present study demonstrates that affinities of the radiocopper-labeled **NODAGA-TATE** variants are similar to those of the clinical reference **[^177^Lu]Lu‑DOTA-TATE** (*K*_d_ = 1-3 nmol/L), with comparisons made under the same experimental settings.

According to the *B*_max_ values measured in the present study, binding capacities of MPC cells for both the copper‑64- and copper‑67-labeled versions of **NODAGA-TATE** and **NODAGA-cLAB4‑TATE** are lower compared to **[^177^Lu]Lu‑DOTA-TATE**. In addition, MPC cells also exhibit lower *B*_max_ values for **[^64^Cu]Cu‑DOTA-TATE**, indicating that this trend is independent of the structural alterations resulting from the replacement of the chelator or the introduction of the albumin-binding cLAB4 domain. So far, explanations are challenging, and possible hypotheses are included in the *Supporting information*.

High efficacy of radioligands in cancer treatment requires high retention within the target tissue. Since radiolabeled TATE variants act as SSTR2 agonists, effective retention in tumor cells initially depends on a high proportion of binding-induced endocytosis of the ligand-receptor-complex. Radioligand uptake in MPC cells indicates that both the copper‑64- and copper‑67-labeled versions of **NODAGA-TATE** and **NODAGA-cLAB4‑TATE** induce SSTR2-mediated endocytosis to a similar extent as **[^177^Lu]Lu‑DOTA-TATE**. Slightly higher uptake fractions have been reported for **[^64^Cu]Cu‑NODAGA-TATE** in genetically modified HTC116-SSTR2+ cells 41.

Beyond cellular uptake, retention of a radioligand in tumors is further determined by its cellular release rate. The present study demonstrates that the release of the copper‑64-labeled versions of **NODAGA-TATE** and **NODAGA-cLAB4‑TATE** from MPC cells is faster compared to **[^177^Lu]Lu‑DOTA-TATE**. Faster release rates, in particular during the late phase of the biphasic process (2-24 h after radioligand removal), suggest that export of the radiocopper-labeled ligands occurs via the endosomal SSTR2 recycling pathway. The release kinetics measured during this phase are largely similar to what has been reported for 'externalization' of **[^64^Cu]Cu‑DOTA-TATE**, **[^68^Ga]Ga‑DOTA-TATE**, and **[^177^Lu]Lu‑DOTA-TATE** from gene-modified HEK293-SSTR2+ cells 42-44. Different from the methodology used in these reports, radioligand release was monitored for 24 hours in the present study, considering the kinetics of both surface-bound and uptake fractions. Time-activity-curves from the present experimental settings indicate that observation for more than three hours is required to reliably detect differences in cellular release rates* in vitro*. In this context, it should be noted that the experimental procedure used in the present study involved repeated removal of the released radioligand fraction from the supernatant to prevent re‑binding and re-uptake.

Hypotheses about faster release of radiocopper-labeled **NODAGA-TATE** variants from tumor cells compared to **[^177^Lu]Lu‑DOTA-TATE** may address possible differences in downstream trafficking and metabolic fate of the ligand-SSTR2 complex. It has been described that the kinetic rates for endosomal recycling of somatostatin receptors vary depending on the chemical structure of the activating agonist 45, 46. Therefore, it can be assumed that the radiocopper-labeled **NODAGA-TATE** variants have a higher preference for endosomal recycling compared to **[^177^Lu]Lu‑DOTA-TATE**.

Upon activation of G‑protein-coupled receptors, a fraction of the ligand-receptor complexes is delivered into the lysosomal pathway for degradation 46. Therefore, specific radiometabolites generated from metabolic conversion of the radiolabeled **NODAGA-TATE** and **DOTA-TATE** variants may determine the release of activity from the cells. For example, the lysosomal fraction of **[^111^In]In‑DTPA-octreotide** is almost completely metabolized to **[^111^In]In‑DTPA‑D‑Phe‑OH** and **[^111^In]In‑DTPA‑D‑Phe‑Cys‑OH.** These radiometabolites are poor substrates for the carrier-mediated transport systems found in lysosomes, thus contributing significantly to intracellular trapping 47. The faster release of radiocopper-labeled **NODAGA-TATE** variants from MPC cells may therefore suggest a higher metabolic stability against lysosomal degradation compared to **[^177^Lu]Lu‑DOTA-TATE** or a different efflux behavior of the respective radiometabolites, resulting in lower amounts of radiometabolites trapped in lysosomes. Nevertheless, the intracellular fate of radiolabeled TATE variants in MPC cells remains to be investigated.

In the present study, a tumor spheroid model of MPC was used to determine the initial concentrations of copper‑67-labeled **NODAGA-TATE** variants enabling adequate uptake and growth inhibition *in vitro*. Growth-reducing effects on spheroids demonstrated that a six‑fold higher initial activity concentration is required for **[^67^Cu]Cu‑NODAGA-TATE** and **[^67^Cu]Cu‑NODAGA-cLAB4-TATE** to provide similar treatment efficacy as **[^177^Lu]Lu‑DOTA-TATE**. This outcome was consistent with lower binding capacities and higher release rates for the copper‑64-labeled versions of the radioligands. These results indicate that effective treatment with copper‑67-labeled **NODAGA-TATE** variants *in vivo* might depend on high initial concentrations and long residence times in tumor tissue to maximize the effective initial uptake and to compensate for faster cellular release.

One approach to increase overall tumor uptake is the incorporation of low-molecular weight albumin binders into radioligands 48. The present study confirmed the effectiveness of this approach, as the albumin binder conjugate **[^67^Cu]Cu‑NODAGA-cLAB4‑TATE** demonstrated higher uptake and retention in MPC tumors compared to **[^67^Cu]Cu‑NODAGA‑TATE**. As previously discovered within a series of **NODAGA-cLAB‑TATE** versions, the moderate albumin binding affinity of **[^64^Cu]Cu‑NODAGA-cLAB4‑TATE** (*K*_d_[HSA] = 50 µmol/L) provided the most favorable pharmacokinetic profile in tumors with highest uptake values and highest retention over 48 h, while other cLAB-conjugates with higher albumin binding affinity were less favorable 24. In this context, the initially higher tumor uptake appears to be surprising, considering that binding to albumin in the blood circulation lowers the actual concentration of the free radioligand. This phenomenon is part of ongoing investigations.

The pharmacokinetic advantages of **[^67^Cu]Cu‑NODAGA-cLAB4‑TATE** provided a four-fold higher cumulated activity and thus absorbed dose in MPC tumors compared to **[^67^Cu]Cu‑NODAGA-TATE**. This effect is lower compared to the approximately 20‑fold improvement facilitated by modification of yttrium‑90- and lutetium‑177-labeled DOTA-TATE with the albumin-affine truncated Evans Blue (EB) dye in other SSTR2-positive tumor models (as calculated from biodistribution data therein) 25, 49. It is also noteworthy that yttrium‑90- and lutetium‑177-labeled **DOTA-EB‑TATE** versions unfold their pharmacokinetic advantages at a higher albumin affinity (*K*_d_[HSA] = 4.8 µmol/L for **DOTA-EB-TATE**) compared to **[^64^Cu]Cu‑NODAGA-cLAB4‑TATE** 24, 25. Unfortunately, recently published data on tumor pharmacokinetics of the albumin-binding **[^67^Cu]Cu‑DOTA-EB‑TATE** in BON1-SSTR2+ and QGP‑1‑SSTR2+ xenograft mice lack a head-to-head comparison with the reference ligand **[^67^Cu]Cu‑DOTA‑TATE** 50.

Survival analyses of MPC tumor-bearing mice demonstrated that treatment with **[^67^Cu]Cu‑NODAGA-cLAB4‑TATE** is approximately 2.5 times more effective compared to **[^67^Cu]Cu‑NODAGA-TATE**. This increase is similar to the range of improvement demonstrated for **[^177^Lu]Lu‑DOTA-EB-TATE** in recent clinical trials, where half of the initial activity dose compared to **[^177^Lu]Lu‑DOTA-TATE** provided equivalent efficacy against SSTR2-positive NENs 15. A recent preclinical study demonstrated equivalent efficacy of **[^67^Cu]Cu‑DOTA-EB-TATE** and **[^177^Lu]Lu‑DOTA-EB‑TATE** against gene-modified BON1-SSTR2+ tumor xenografts in mice 50. In the future, it has to be investigated whether the kinetic inertness of copper-DOTA complexes (transchelation) is compatible with long blood retention of **[^67^Cu]Cu‑DOTA-EB-TATE** in clinical settings.

In addition to ligand-specific pharmacokinetic profiles in tumors, physical half-life of the radionuclide determines the time course of effective activity concentrations and thus the therapy-relevant absorbed dose. Despite a shorter physical half-life of copper‑67 compared to lutetium‑177, treatment with **[^67^Cu]Cu‑NODAGA-cLAB4‑TATE** provided similar absorbed doses and growth-reducing effects in MPC tumors as **[^177^Lu]Lu‑DOTA-TATE**. The highest absorbed doses in MPC tumors per administered activity of **[^67^Cu]Cu‑NODAGA-cLAB4‑TATE** and **[^177^Lu]Lu‑DOTA-TATE** (0.80-0.82 Gy/MBq) are higher compared to other naturally SSTR2-positive neuroendocrine tumor models in mice treated with **[^177^Lu]Lu‑DOTA-TATE**, for example NCI‑H69 xenografts (0.33-0.36 Gy/MBq) and GOT‑1 xenografts (0.27 Gy) 51-53. Nevertheless, growth-delaying effects from treatment with **[^177^Lu]Lu‑DOTA-TATE** were stronger even at lower total absorbed doses in NCI‑H69 xenografts (10.0-10.2 Gy) and GOT‑1 xenografts (8.1 Gy) compared to MPC tumors in the present study (22.0-41.1 Gy) 51-53, indicating that MPC tumor allografts exhibit a more radioresistant phenotype.

Due to the higher initial activity concentrations in tumors followed by shorter effective tissue half-life, **[^67^Cu]Cu‑NODAGA-cLAB4‑TATE** deposits its therapeutic dose in a shorter time and thus with a higher dose rate compared to **[^177^Lu]Lu‑DOTA-TATE**. From a radiobiologic standpoint, higher dose rates delivered over shorter times have been suggested to be more effective than lower dose rates delivered over longer periods. Thus, radionuclides with a shorter half-life are considered to be more biologically effective than those with a similar emission energy but longer half-life 19. Equivalent effects of **[^67^Cu]Cu‑NODAGA-cLAB4‑TATE** and **[^177^Lu]Lu‑DOTA-TATE** on the survival of MPC tumor-bearing mice may not fully support this hypothesis. On the other hand, a high initial activity concentration and faster effective half-life in tumors may be favorable for fractionated therapies since the duration of low-dose-exposure is relatively short, potentially limiting the evolution of tumor cells with a radioresistant phenotype.

For metalated radiotherapeutics, a high kinetic inertness is required *in vivo* to avoid the release of free radiometal ions that accumulate in non-target organs. Free radiocopper ions preferentially accumulate in the liver followed by a slow elimination 54. For radiocopper-labeled targeting vectors with a generally low liver avidity, kinetic inertness can therefore be estimated from the activity profiles in the liver. A low uptake in the mouse liver indicates that copper-NODAGA complexes provide favorable stability for theranostic application of copper‑67-labeled SSTR2 radioligands, including those with slow blood kinetics. This is consistent with previous preclinical reports on long-circulating radiocopper-labeled NODAGA-immunoconjugates demonstrating improved complex stability compared to their DOTA versions in mice 29. Besides, the activity concentrations detected in mouse liver one hour after treatment with **[^67^Cu]Cu‑NODAGA-cLAB4‑TATE** (4.10 % ID/mL) are substantially lower compared to what has been reported for **[^67^Cu]Cu‑DOTA-EB‑TATE** (10.8 %ID/g) 50. As documented therein, further increase in liver activity three days after treatment with **[^67^Cu]Cu‑DOTA-EB‑TATE** (14.6 %ID/g) indicates the release of [^67^Cu]Cu^2+^ from the DOTA complex, which does not occur after treatment with **[^67^Cu]Cu‑NODAGA-cLAB4‑TATE**. Early occurrence of activity in the liver followed by continuous elimination suggests that the prolonged blood retention of **[^67^Cu]Cu‑NODAGA-cLAB4-TATE** as well as free residual [^67^Cu]Cu^2+^ ions (< 4%) in the radioligand formulations most likely contributed to the cumulated activity. This stresses the necessity for high radiochemical purities in the formulation of copper‑67-labeled SSTR2 radioligands for theranostic applications* in vivo*.

Apart from the kinetic inertness of the copper-NODAGA-complex, the metabolic stability of the target molecules themselves need to be considered for *in vivo* application. Previously, we characterized **[^64^Cu]Cu‑NODAGA-TATE** and **[^64^Cu]Cu‑NODAGA-cLAB4‑TATE** for their metabolic stability in mouse and human plasma *in vitro* for up to 24 hours 24. **[^64^Cu]Cu‑NODAGA-TATE** exhibited an excellent stability (half-life > 24 h, > 75 % residual intact radioligand), while **[^64^Cu]Cu‑NODAGA-cLAB4‑TATE** was rather rapidly metabolized to one distinct radiometabolite (half-life of ≈ 3 h). This radiometabolite originated from hydrolysis of the primary amide functionality within the cLAB4 domain to the respective carboxylic acid due to the action of plasma carboxylesterases. It is worth noting that this metabolic transformation is accompanied by an increase in albumin binding affinity, which in turn might affect the overall biodistribution. Although we previously speculated that this metabolization barely affects the tumor uptake curve, the potential consequences need to be further investigated, which is part of ongoing studies by us. In contrast, we did not observe the amide hydrolysis in human plasma for **[^64^Cu]Cu-NODAGA-cLAB4-TATE** as in human plasma the activity of plasma carboxylesterases is only minimal (if present at all). This is probably the most important aspect for the potential radiotherapeutic application of **[^67^Cu]Cu-NODAGA-cLAB4-TATE**.

During renal excretion, peptides are subject to proximal tubular re-absorption and thus retention in kidneys 55. Both **[^67^Cu]Cu‑NODAGA-cLAB4-TATE** and **[^67^Cu]Cu‑NODAGA-TATE** are characterized by higher initial uptake in kidneys compared to **[^177^Lu]Lu‑DOTA-TATE**. This has also been observed with other radiolabeled peptides, including **[^64^Cu]Cu‑SARTATE**, suggesting that the effect is related to the net charge of the conjugates 41, 56, 57. It should be noted that all treatments in the present study were performed without kidney-protective measures. This is different from the clinical routine, where basic amino acids are typically infused before, during, and after PRRT to reduce re-absorption of the radiolabeled peptides and thus the radiation dose in kidneys 58. In the present study, binding of **[^67^Cu]Cu‑NODAGA-cLAB4-TATE** to albumin improved the tumor-to-kidney dose ratio compared to treatment with **[^67^Cu]Cu‑NODAGA-TATE**.

In PRRT, increasing proportions of non-radiolabeled peptides in the radioligand preparation are typically associated with reduced uptake in target tissues 59, 60. For treatments with identical activity doses, higher peptide amounts are administered as the molar activity of the radioligand preparation decreases. As expected, a reduction in molar activity from 40 to 20 MBq/nmol also reduced the initial uptake of the copper‑67-labeled **NODAGA-TATE** variants in MPC tumors. This effect may be caused by increasing saturation of accessible SSTR2 binding sites by higher amounts of competing non-radiolabeled peptide. Interestingly, copper‑67-labeled **NODAGA-TATE** variants maintained most of their therapeutic efficacy even at the lower molar activity of 20 MBq/nmol, in contrast to a more pronounced decline in the therapeutic efficacy of **[^177^Lu]Lu‑DOTA-TATE**. Typically, tumor uptake of radiolabeled SSTR2 antagonists shows higher robustness against increasing peptide amounts, as tumor cells have a much higher binding capacity for antagonists compared to agonists 42, 59, 61. However, the opposite was discovered for the binding capacities of MPC cells for radiocopper‑labeled **NODAGA-TATE** variants. Therefore, it can be hypothesized that the pharmacological behavior of the non-radiolabeled **NODAGA-TATE** variants *in vivo* prevents them from fully exerting their competitive effects on the radiolabeled peptide fraction.

In peptide receptor radionuclide therapy, red bone marrow and kidneys are the two main dose-limiting organs 62, 63. Therefore, hematotoxicity and nephrotoxicity were of particular concern in the present study. Rapid normalization of leukocyte counts in mice within 20 days after treatment indicate that the copper‑67-labeled **NODAGA-TATE** variants are well tolerated with only mild and reversible hematologic adverse effects. In particular, the substantially higher cumulated activity in blood after treatment with **[^67^Cu]Cu‑NODAGA-cLAB4‑TATE** did not translate into higher leukocyte toxicity. This is consistent with a low incidence of hematotoxicity reported for treatments with the albumin-binding radioligand **[^177^Lu]Lu‑DOTA-EB‑TATE** exhibiting an even longer effective half‑life in blood 15. SSTR2 are present on a small subset (1 %) of red bone marrow cells, including pluripotent stem cells and early lineage-committed progenitor cells 64. It can therefore be assumed that specific uptake and effective half-lives of the therapeutic SSTR2 radioligands in these hematopoietic cell populations, rather than blood retention alone, determine leukocyte toxicity.

Moderately increasing renal albumin excretion in mice within 20 days after treatment indicates that copper‑67-labeled **NODAGA-TATE** variants have some effect on kidney function, which is however tolerated without deterioration in general condition. This is in line with a preclinical study on the renal effects of **[^177^Lu]Lu‑DOTA-TATE** in nude mice demonstrating that treatment with a single activity dose of 90 MBq is already associated with detectable tubular damage in tissue samples and increased serum creatinine, while body weights remain stable 55. The same study demonstrated a dose-response relationship for proximal tubular damage with a threshold dose value of 24 Gy absorbed in the renal cortex. In mice, the tissue weight of the renal cortex can be estimated as 60 % of total kidney weight 55. Assuming that the measured total kidney dose is solely absorbed by the renal cortex, the estimated dose value for treatment with **[^67^Cu]Cu‑NODAGA-cLAB4‑TATE** is approximately 9.5 Gy, which is well below the proposed threshold value.

Investigations on renal function in the present study represent a short-term follow-up owing to the limited survival time of the tumor-bearing mice. Other preclinical studies in mice demonstrate that functional impairment of kidneys can persist for more than 24 weeks after PRRT 55, 65. Therefore, possible long-term effects on renal function from treatments with **[^67^Cu]Cu‑NODAGA-TATE** and **[^67^Cu]Cu‑NODAGA-cLAB4-TATE** cannot be excluded. However, clinical experience from treatments with **[^177^Lu]Lu‑DOTA-TATE** and the albumin binder conjugate **[^177^Lu]Lu‑DOTA-EB-TATE** indicate that total absorbed kidney doses of up to 23 Gy are well tolerated without acute nephrotoxicity of any grade 15, 63, 66.

Dosimetry models using preclinical small animal imaging data are commonly employed to predict the radiation dose that would be experienced by human subjects prior to clinical application of a radioactive agent 36, 67, 68. The present study demonstrates that *in vivo* dosimetry for treatments with copper‑67-labeled **NODAGA-TATE** variants in mice benefits form higher count rates and spatial resolution in SPECT imaging compared to **[^177^Lu]Lu‑DOTA-TATE**. As predicted from the extrapolated pharmacokinetic profiles in mice, the projected human organ doses for treatment with **[^67^Cu]Cu‑NODAGA-cLAB4‑TATE** are within an acceptable range for clinical application. This applies in particular to dose-limiting organs. For example, projected female doses in bone marrow and kidneys for treatment with **[^67^Cu]Cu‑NODAGA-cLAB4‑TATE** are 17‑fold and 1.6-fold higher, respectively, compared to **[^177^Lu]Lu‑DOTA-TATE**. Besides, clinical application of the albumin-binding conjugate **[^177^Lu]Lu‑DOTA-EB‑TATE** compared to **[^177^Lu]Lu‑DOTA-TATE** is associated with up to 19‑fold and 3.2‑fold higher doses in bone marrow and kidneys, respectively 16. It should also be noted that the human kidney doses predicted in the present study do not take renal protection into account.

Regarding the accuracy of the human dose projections herein, the effective doses in males and females for treatment with **[^177^Lu]Lu‑DOTA-TATE** (0.024-0.031 mSv/MBq) are largely consistent with formerly published results based on preparative biodistribution data in mice (0.032 mSv/MBq) 59. Nevertheless, extrapolation from preclinical data underestimates the human effective doses determined for **[^177^Lu]Lu‑DOTA-TATE** in clinical dosimetry (0.069 mSv/MBq) 16. This is consistent with a reported underestimation of 38-47 % for human effective doses extrapolated from animal PET imaging data of fluorine‑18-labeled radiotracers, although the kinetic profiles were scaled with respect to mass and time scale differences between species 36. Taking this underestimation into account, an assumed 1.9‑fold higher human effective dose resulting from treatment with **[^67^Cu]Cu‑NODAGA-cLAB4‑TATE** (0.14 mSv/MBq) would be in the same range as reported for **[^177^Lu]Lu‑DOTA-EB‑TATE** (0.08-0.14 mSv/MBq) 16, 69.

## Conclusion

The presented preclinical study demonstrates fundamental therapeutic efficacy of **[^67^Cu]Cu‑NODAGA-cLAB4‑TATE** in SSTR2-positive tumors. As an intrinsic radionuclide theranostic agent, the radioligand offers stable copper‑67 complexes and high sensitivity in SPECT imaging for prospective determination and monitoring of therapy efficacy *in vivo*. In addition, copper‑64- and copper‑61-labeled versions offer alternative options for pre- and post-therapeutic PET. Therefore, **NODAGA-cLAB4-TATE** has the potential to advance clinical use of radiocopper in SSTR2 targeted cancer theranostics. Prospectively, alternative copper chelators and/or charge-modulating structural modifications may further improve tumor retention.

## Supplementary Material

Supplementary materials and methods, figures and tables.

## Figures and Tables

**Table 1:** SSTR2 binding constants and uptake fractions of gallium‑68-, lutetium‑177-, copper‑64-, and copper‑67-labeled TATE variants measured in intact MPC cells

TATE variant	RN	*A*_m_	*K*_d_ *^ a^*	*B*_max_ *^ a^*		Uptake fraction*^ b^*	
		[MBq/nmol]	[nmol/L]	[pmol/mg protein]	*n^ c^*	[%]	*n^ d^*
DOTA	^68^Ga	26.8 ± 0.27	2.92 ± 0.78	1.18 ± 0.39	4	87 ± 1.9	3
	^177^Lu	26.7 ± 1.17	2.94 ± 0.52	1.21 ± 0.24	5	73 ± 2.2	5
	^64^Cu	23.2 ± 1.35	0.97 ± 0.17 *^†^*	0.46 ± 0.05 *	8	61 ± 4.6	5
NODAGA	^64^Cu	25.0 ± 0.97	1.78 ± 0.34	0.47 ± 0.04 *	6	64 ± 4.1	6
	^67^Cu	18.2	2.55 (2.12-2.98)	0.82 (0.79-0.85)	1	54 (52-55)	1
NODAGA-cLAB4	^64^Cu	26.7 ± 0.71	2.72 ± 0.52	0.60 ± 0.08	4	72 ± 6.3	3
	^67^Cu	24.0	2.74 (1.90-3.59)	0.61 (0.56-0.66)	1	61 (59-62)	1

(*A*_m_) molar activity at incubation start; (RN) radionuclide; *^a^* binding constants were measured at free radioligand concentrations between 0.3-40 nmol/L; non-specific binding was assessed in presence of 1 nmol/L acetyl-TATE; *^b^* non-acid-releasable radioligand fraction, values normalized to specifically bound radioligand after initial exposure to a free concentration of 25 nmol/L; number of independent experiments (*n*) each performed in*^ c^* triplicate or *^d^* sextuplicate; data presented as means ± standard error (*n* > 3) or confidence interval of 68 % (*n* < 3); significance of differences compared to **[^177^Lu]Lu‑DOTA-TATE**: * *p* < 0.05, *^†^ p* < 0.01

**Table 2:** Release of lutetium‑177- and copper‑64-labeled TATE variants from intact MPC cells

TATE variant	RN	*A*_m_	Fast fraction^*a*^	*t*_1/2_ (fast)	*t*_1/2_ (slow)	24 h release^*a*^	Exp.
		[MBq/nmol]	[%]	[hours]	[hours]	[%]	*n^ b^*
DOTA	^177^Lu	37.9 ± 7.42	39 ± 3.9	0.82 ± 0.19	68.0 ± 25.1	52 ± 1.9	4
NODAGA	^64^Cu	44.7 ± 7.98	42 ± 3.3	0.74 ± 0.13	21.4 ± 2.19*^ †^*	73 ± 1.9^ #^	5
NODAGA-cLAB4	^64^Cu	42.5 ± 9.15	45 ± 1.4	0.52 ± 0.12	20.0 ± 3.44*^ †^*	76 ± 2.0^ #^	4

(*A*_m_) molar activity at incubation start; (RN) radionuclide; *^a^* relative loss of radioligand normalized to initial SSTR2-specific binding after exposure to the radioligand (25 nmol/L) for 1 hour; *^b^* number of independent experiments (*n*) each performed in sextuplicate; data presented as means ± standard error; significance of differences compared to **[^177^Lu]Lu‑DOTA-TATE**: *^†^ p* < 0.01, *^#^ p* < 0.001

**Table 3:** Absorbed doses in MPC tumor allografts and animal survival after treatment with lutetium‑177- and copper‑67-labeled TATE variants

TATE variant	RN	*A*_m_	*D_T,_*_Tumor_	PFS ^#^	OS^*†*^	Animals
		[MBq/nmol]	[Gy]	[days]	[days]	*n* (per exp.)
Control/vehicle	-	-	-	3.40	15.2	8 (3, 3, 2)
DOTA	^177^Lu	39.6 ± 0.09	41.1 ± 3.65	18.7	28.4	5 (5)
		22.6 ± 1.42	22.0 ± 1.11	11.3	22.7	6 (3, 3)
NODAGA	^67^Cu	40.3 ± 0.76	9.51 ± 0.95	8.06	21.0	6 (4, 2)
		24.6 ± 0.67	7.82 ± 0.79	7.42	16.9	5 (3, 2)
NODAGA-cLAB4	^67^Cu	41.5 ± 0.56	40.2 ± 3.09	16.6	27.3	6 (3, 3)
		21.5 ± 0.54	34.6 ± 2.73	13.9	25.2	5 (2, 1, 2)

Each animal received an initial activity dose of 50 MBq; (*A*_m_) molar activity at treatment start; (*D_T_*_, Tumor_) mean absorbed dose in tumors; (*n*) number of animals per group and per independent experiment; (OS) overall survival defined as < 10‑fold change in tumor volume and (PFS) progression-free survival defined as < 2‑fold change in tumor volume compared to treatment start; (RN) radionuclide; *A*_m_, ID, and *D_T_* presented as means ± standard error, PFS and OS presented as medians; Pearson correlation coefficient (*r*_p_) and significance of linear relationships with *D*_T_: *^†^ r* = 0.96, *p* < 0.01, *^#^ r* = 0.98, *p* < 0.001

**Table 4:** Absorbed doses in kidneys and incidence of microalbuminuria after treatment with lutetium‑177- and copper‑67-labeled TATE variants

TATE variant	RN	*A*_m_ *^a^*	*D_T_*_, Kidneys_ *^a^*	Day 6*^ a^*		Day 20*^ a^*	
				MA-positives*^ b^*	Samples*^ c^*	MA-positives*^ b^*	Samples*^ c^*
		[MBq/nmol]	[Gy]	[*n*/samples] (%)	[*n*/cohort]	[*n*/samples] (%)	[*n*/cohort]
Control/vehicle	-	-	-	1/7 (14)	7/8	-	-
DOTA	^177^Lu	39.6 ∪ 22.6	2.81 ± 0.14	1/6 (17)	6/11	1/3 (33)	3/11
NODAGA	^67^Cu	40.3 ∪ 24.6	2.42 ± 0.20	2/6 (33)	6/11	3/4 (75)	4/11
NODAGA-cLAB4	^67^Cu	41.5 ∪ 21.5	6.82 ± 0.40	2/8 (25)	8/11	4/8 (50)	8/11

(*A*_m_) molar activity at treatment start; (*D*_T, Kidneys_) mean absorbed dose in kidneys ± standard error; (MA) microalbuminuria; (RN) radionuclide; *^a^* pooled data from treatments at molar activities of 40 and 20 MBq/nmol; *^b^* proportion of urine samples showing microalbuminuria at ACR > 30 < 300 mg/g; *^c^* proportion of the total cohort providing urine samples suitable for analysis; urine samples could not be analyzed when creatinine and/or albumin concentrations were below detection limits; no urine samples were available from animals withdrawn from follow-up before reaching the indicated sampling point

## Abbreviations

*A*_m_: molar activity
ACR: albumin-creatinine-ratio
cLAB: “clickable” lysine-derived albumin binder
CT: X-ray computed tomography
EB: Evans Blue
MPC: mouse pheochromocytoma
NENs: neuroendocrine neoplasms
OS: overall survival
PCC/PGL: pheochromocytoma and paraganglioma
PET: positron emission tomography
PFS: progression-free survival
PRRT: peptide receptor radionuclide therapy
SPECT: single-photon emission computed tomography
SSTR2: somatostatin type 2 receptor
TATE: (Tyr^3^)octreotate

## References

[1] Basu S, Parghane RV, Kamaldeep, Chakrabarty S (2020). Peptide receptor radionuclide therapy of neuroendocrine tumors. Semin Nucl Med.

[2] Nolting S, Ullrich M, Pietzsch J, Ziegler CG, Eisenhofer G, Grossman A (2019). Current management of pheochromocytoma/paraganglioma: a guide for the practicing clinician in the era of precision medicine. Cancers (Basel).

[3] Taieb D, Wanna GB, Ahmad M, Lussey-Lepoutre C, Perrier ND, Nolting S (2023). Clinical consensus guideline on the management of phaeochromocytoma and paraganglioma in patients harbouring germline SDHD pathogenic variants. Lancet Diabetes Endocrinol.

[4] Leijon H, Remes S, Hagstrom J, Louhimo J, Maenpaa H, Schalin-Jantti C (2019). Variable somatostatin receptor subtype expression in 151 primary pheochromocytomas and paragangliomas. Hum Pathol.

[5] Fischer A, Kloos S, Maccio U, Friemel J, Remde H, Fassnacht M (2023). Metastatic pheochromocytoma and paraganglioma: somatostatin receptor 2 expression, genetics, and therapeutic responses. J Clin Endocrinol Metab.

[6] Parghane RV, Naik C, Talole S, Desmukh A, Chaukar D, Banerjee S (2020). Clinical utility of ^177^Lu-DOTATATE PRRT in somatostatin receptor-positive metastatic medullary carcinoma of thyroid patients with assessment of efficacy, survival analysis, prognostic variables, and toxicity. Head Neck.

[7] Marretta AL, Ottaiano A, Iervolino D, Bracigliano A, Clemente O, Di Gennaro F (2023). Response to peptide receptor radionuclide therapy in pheocromocytomas and paragangliomas: a systematic review and meta-analysis. J Clin Med.

[8] Clement D, Navalkissoor S, Srirajaskanthan R, Courbon F, Dierickx L, Eccles A (2022). Efficacy and safety of ^177^Lu-DOTATATE in patients with advanced pancreatic neuroendocrine tumours: data from the NETTER-R international, retrospective study. Eur J Nucl Med Mol Imaging.

[9] ICRP 2008 Nuclear decay data for dosimetric calculations. ICRP Publication 107. Ann. ICRP 38 (3).

[10] Sjogreen Gleisner K, Chouin N, Gabina PM, Cicone F, Gnesin S, Stokke C (2022). EANM dosimetry committee recommendations for dosimetry of ^177^Lu-labelled somatostatin-receptor- and PSMA-targeting ligands. Eur J Nucl Med Mol Imaging.

[11] Strosberg J, El-Haddad G, Wolin E, Hendifar A, Yao J, Chasen B (2017). Phase 3 trial of ^177^Lu-DOTATATE for midgut neuroendocrine tumors. N Engl J Med.

[12] Deshayes E, Assenat E, Meignant L, Bardies M, Santoro L, Gourgou S (2022). A prospective, randomized, phase II study to assess the schemas of retreatment with Lutathera in patients with new progression of an intestinal, well-differentiated neuroendocrine tumor (ReLUTH). BMC Cancer.

[13] Wolf KI, Jha A, van Berkel A, Wild D, Janssen I, Millo CM (2019). Eruption of metastatic paraganglioma after successful therapy with ^177^Lu/^90^Y-DOTATOC and ^177^Lu-DOTATATE. Nucl Med Mol Imaging.

[14] Sandstrom M, Garske-Roman U, Granberg D, Johansson S, Widstrom C, Eriksson B (2013). Individualized dosimetry of kidney and bone marrow in patients undergoing ^177^Lu-DOTA-octreotate treatment. J Nucl Med.

[15] Jiang Y, Liu Q, Wang G, Sui H, Wang R, Wang J (2022). Safety and efficacy of peptide receptor radionuclide therapy with ^177^Lu-DOTA-EB-TATE in patients with metastatic neuroendocrine tumors. Theranostics.

[16] Zhang J, Wang H, Jacobson O, Cheng Y, Niu G, Li F (2018). Safety, pharmacokinetics, and dosimetry of a long-acting radiolabeled somatostatin analog ^177^Lu-DOTA-EB-TATE in patients with advanced metastatic neuroendocrine tumors. J Nucl Med.

[17] Hehakaya C, Moors EHM, Verkooijen HM, Grobbee DE, Verburg FA, Lam M (2021). ^177^Lu-PSMA for advanced prostate cancer: are we ready to play big?. Eur J Nucl Med Mol Imaging.

[18] Vogel WV, van der Marck SC, Versleijen MWJ (2021). Challenges and future options for the production of lutetium-177. Eur J Nucl Med Mol Imaging.

[19] Kassis AI (2008). Therapeutic radionuclides: biophysical and radiobiologic principles. Semin Nucl Med.

[20] Levart D, Kalogianni E, Corcoran B, Mulholland N, Vivian G (2019). Radiation precautions for inpatient and outpatient ^177^Lu-DOTATATE peptide receptor radionuclide therapy of neuroendocrine tumours. EJNMMI Phys.

[21] Hao G, Mastren T, Silvers W, Hassan G, Oz OK, Sun X (2021). Copper-67 radioimmunotheranostics for simultaneous immunotherapy and immuno-SPECT. Sci Rep.

[22] Deppen SA, Liu E, Blume JD, Clanton J, Shi C, Jones-Jackson LB (2016). Safety and efficacy of ^68^Ga-DOTATATE PET/CT for diagnosis, staging, and treatment management of neuroendocrine tumors. J Nucl Med.

[23] Stenvall A, Gustafsson J, Larsson E, Roth D, Sundlov A, Jonsson L (2022). Relationships between uptake of [^68^Ga]Ga-DOTA-TATE and absorbed dose in [^177^Lu]Lu-DOTA-TATE therapy. EJNMMI Res.

[24] Brandt F, Ullrich M, Laube M, Kopka K, Bachmann M, Loser R (2022). "Clickable" albumin binders for modulating the tumor uptake of targeted radiopharmaceuticals. J Med Chem.

[25] Tian R, Jacobson O, Niu G, Kiesewetter DO, Wang Z, Zhu G (2018). Evans Blue attachment enhances somatostatin receptor subtype-2 imaging and radiotherapy. Theranostics.

[26] Morgan KA, Donnelly PS (2021). Chapter Two - Metallic radionuclides for diagnostic imaging and cancer radiotherapy: The development of theragnostic matched pairs and targeted alpha therapy. In: Hubbard CD, van Eldik R, editors. Advances in Inorganic Chemistry: Academic Press.

[27] Krasnovskaya OO, Abramchuck D, Erofeev A, Gorelkin P, Kuznetsov A, Shemukhin A (2023). Recent advances in ^64^Cu/^67^Cu-based radiopharmaceuticals. Int J Mol Sci.

[28] Bruhlmann SA, Walther M, Kreller M, Reissig F, Pietzsch HJ, Kniess T (2023). Cyclotron-based production of ^67^Cu for radionuclide theranostics via the ^70^Zn(p,alpha)^67^Cu reaction. Pharmaceuticals (Basel).

[29] Ghosh SC, Pinkston KL, Robinson H, Harvey BR, Wilganowski N, Gore K (2015). Comparison of DOTA and NODAGA as chelators for ^64^Cu-labeled immunoconjugates. Nucl Med Biol.

[30] Ullrich M, Bergmann R, Peitzsch M, Cartellieri M, Qin N, Ehrhart-Bornstein M (2014). *In vivo* fluorescence imaging and urinary monoamines as surrogate biomarkers of disease progression in a mouse model of pheochromocytoma. Endocrinology.

[31] Kreller M, Pietzsch HJ, Walther M, Tietze H, Kaever P, Knieß T (2019). Introduction of the new Center for Radiopharmaceutical Cancer Research at Helmholtz-Zentrum Dresden-Rossendorf. Instruments.

[32] Thieme S, Walther M, Pietzsch HJ, Henniger J, Preusche S, Mading P (2012). Module-assisted preparation of ^64^Cu with high specific activity. Appl Radiat Isot.

[33] Ullrich M, Bergmann R, Peitzsch M, Zenker EF, Cartellieri M, Bachmann M (2016). Multimodal somatostatin receptor theranostics using [^64^Cu]Cu-/[^177^Lu]Lu-DOTA-(Tyr^3^)octreotate and AN-238 in a mouse pheochromocytoma model. Theranostics.

[34] Ullrich M, Brandt F, Loser R, Pietzsch J, Wodtke R (2023). Comparative saturation binding analysis of ^64^Cu-labeled somatostatin analogues using cell homogenates and intact cells. ACS Omega.

[35] Keenan MA, Stabin MG, Segars WP, Fernald MJ (2010). RADAR realistic animal model series for dose assessment. J Nucl Med.

[36] Kranz M, Sattler B, Tiepolt S, Wilke S, Deuther-Conrad W, Donat CK (2016). Radiation dosimetry of the alpha_4_beta_2_ nicotinic receptor ligand (+)-[^18^F]flubatine, comparing preclinical PET/MRI and PET/CT to first-in-human PET/CT results. EJNMMI Phys.

[37] A.P (2016). Breeman W, Sze Chan H, M.S. de Zanger R, K. Konijnenberg M, de Blois E. Overview of development and formulation of ^177^Lu-DOTA-TATE for PRRT. Current Radiopharmaceuticals.

[38] Nanabala R, Pillai MRA, Gopal B (2022). Preparation of patient doses of [^177^Lu]Lu-DOTATATE and [^177^Lu]Lu-PSMA-617 with carrier added (CA) and no carrier added (NCA) ^177^Lu. Nucl Med Mol Imaging.

[39] Ullrich M, Liers J, Peitzsch M, Feldmann A, Bergmann R, Sommer U (2018). Strain-specific metastatic phenotypes in pheochromocytoma allograft mice. Endocrine-Related Cancer.

[40] Brandt F, Ullrich M, Wodtke J, Kopka K, Bachmann M, Loser R (2023). Enzymological characterization of ^64^Cu-labeled neprilysin substrates and theirapplication for modulating the renal clearance of targeted radiopharmaceuticals. J Med Chem.

[41] Nedrow JR, White AG, Modi J, Nguyen K, Chang AJ, Anderson CJ (2014). Positron emission tomographic imaging of copper 64- and gallium 68-labeled chelator conjugates of the somatostatin agonist tyr3-octreotate. Mol Imaging.

[42] Rylova SN, Stoykow C, Del Pozzo L, Abiraj K, Tamma ML, Kiefer Y (2018). The somatostatin receptor 2 antagonist ^64^Cu-NODAGA-JR11 outperforms ^64^Cu-DOTA-TATE in a mouse xenograft model. PLoS One.

[43] Mansi R, Abid K, Nicolas GP, Del Pozzo L, Grouzmann E, Fani M (2020). A new (68)Ga-labeled somatostatin analog containing two iodo-amino acids for dual somatostatin receptor subtype 2 and 5 targeting. EJNMMI Res.

[44] Mansi R, Plas P, Vauquelin G, Fani M (2021). Distinct *in vitro* binding profile of the somatostatin receptor subtype 2 antagonist [^177^Lu]Lu-OPS201 compared to the agonist [^177^Lu]Lu-DOTA-TATE. Pharmaceuticals (Basel).

[45] Antunes P, Ginj M, Walter MA, Chen J, Reubi JC, Maecke HR (2007). Influence of different spacers on the biological profile of a DOTA-somatostatin analogue. Bioconjug Chem.

[46] Koenig JA (2004). Assessment of receptor internalization and recycling. Methods Mol Biol.

[47] Bass LA, Lanahan MV, Duncan JR, Erion JL, Srinivasan A, Schmidt MA (1998). Identification of the soluble *in vivo* metabolites of indium-111-diethylenetriaminepentaacetic acid-D-Phe1-octreotide. Bioconjug Chem.

[48] Brandt M, Cardinale J, Giammei C, Guarrochena X, Happl B, Jouini N (2019). Mini-review: Targeted radiopharmaceuticals incorporating reversible, low molecular weight albumin binders. Nucl Med Biol.

[49] Bandara N, Jacobson O, Mpoy C, Chen X, Rogers BE (2018). Novel structural modification based on Evans Blue dye to improve pharmacokinetics of a somastostatin-receptor-based theranostic agent. Bioconjug Chem.

[50] Njotu FN, Ketchemen JP, Tikum AF, Babeker H, Gray BD, Pak KY (2024). Efficacy of [^67^Cu]Cu-EB-TATE theranostic against somatostatin receptor subtype-2-positive neuroendocrine tumors. J Nucl Med.

[51] Dalmo J, Spetz J, Montelius M, Langen B, Arvidsson Y, Johansson H (2017). Priming increases the anti-tumor effect and therapeutic window of ^177^Lu-octreotate in nude mice bearing human small intestine neuroendocrine tumor GOT1. EJNMMI Res.

[52] Dalm SU, Nonnekens J, Doeswijk GN, de Blois E, van Gent DC, Konijnenberg MW (2016). Comparison of the therapeutic response to treatment with a ^177^Lu-labeled somatostatin receptor agonist and antagonist in preclinical models. J Nucl Med.

[53] Feijtel D, Doeswijk GN, Verkaik NS, Haeck JC, Chicco D, Angotti C (2021). Inter and intra-tumor somatostatin receptor 2 heterogeneity influences peptide receptor radionuclide therapy response. Theranostics.

[54] Parmar A, Pascali G, Voli F, Lerra L, Yee E, Ahmed-Cox A (2018). *In vivo* [^64^Cu]CuCl_2_ PET imaging reveals activity of Dextran-Catechin on tumor copper homeostasis. Theranostics.

[55] Svensson J, Molne J, Forssell-Aronsson E, Konijnenberg M, Bernhardt P (2012). Nephrotoxicity profiles and threshold dose values for [^177^Lu]-DOTATATE in nude mice. Nucl Med Biol.

[56] Paterson BM, Roselt P, Denoyer D, Cullinane C, Binns D, Noonan W (2014). PET imaging of tumours with a ^64^Cu labeled macrobicyclic cage amine ligand tethered to Tyr^3^-octreotate. Dalton Trans.

[57] Akizawa H, Arano Y, Mifune M, Iwado A, Saito Y, Mukai T (2001). Effect of molecular charges on renal uptake of ^111^In-DTPA-conjugated peptides. Nucl Med Biol.

[58] Hope TA, Abbott A, Colucci K, Bushnell DL, Gardner L, Graham WS (2019). NANETS/SNMMI procedure standard for somatostatin receptor-based peptide receptor radionuclide therapy with ^177^Lu-DOTATATE. J Nucl Med.

[59] Nicolas GP, Mansi R, McDougall L, Kaufmann J, Bouterfa H, Wild D (2017). Biodistribution, pharmacokinetics, and dosimetry of ^177^Lu-, ^90^Y-, and ^111^In-labeled somatostatin receptor antagonist OPS201 in comparison to the agonist ^177^Lu-DOTATATE: the mass effect. J Nucl Med.

[60] Siebinga H, Veerman C, de Wit-van der Veen L, Stokkel MPM, Hendrikx J, Aalbersberg EA (2022). Somatostatin receptor saturation after administration of high peptide amounts of [^177^Lu]Lu-HA-DOTATATE: when enough is enough. EJNMMI Res.

[61] Fani M, Nicolas GP, Wild D (2017). Somatostatin receptor antagonists for imaging and therapy. J Nucl Med.

[62] Bergsma H, Konijnenberg MW, Kam BL, Teunissen JJ, Kooij PP, de Herder WW (2016). Subacute haematotoxicity after PRRT with ^177^Lu-DOTA-octreotate: prognostic factors, incidence and course. Eur J Nucl Med Mol Imaging.

[63] Bergsma H, Konijnenberg MW, van der Zwan WA, Kam BL, Teunissen JJ, Kooij PP (2016). Nephrotoxicity after PRRT with ^177^Lu-DOTA-octreotate. Eur J Nucl Med Mol Imaging.

[64] Oomen SP, Hofland LJ, van Hagen PM, Lamberts SW, Touw IP (2000). Somatostatin receptors in the haematopoietic system. Eur J Endocrinol.

[65] Kristiansson A, Ahlstedt J, Holmqvist B, Brinte A, Tran TA, Forssell-Aronsson E (2019). Protection of kidney function with human antioxidation protein alpha_1_ microglobulin in a mouse ^177^Lu-DOTATATE radiation therapy model. Antioxid Redox Signal.

[66] Hanscheid H, Hartrampf PE, Schirbel A, Buck AK, Lapa C (2021). Intraindividual comparison of [^177^Lu]Lu-DOTA-EB-TATE and [^177^Lu]Lu-DOTA-TOC. Eur J Nucl Med Mol Imaging.

[67] Garrow AA, Andrews JPM, Gonzalez ZN, Corral CA, Portal C, Morgan TEF (2020). Preclinical dosimetry models and the prediction of clinical doses of novel positron emission tomography radiotracers. Sci Rep.

[68] Ling X, Latoche JD, Choy CJ, Kurland BF, Laymon CM, Wu Y (2020). Preclinical dosimetry, imaging, and targeted radionuclide therapy studies of Lu-177-labeled albumin-binding, PSMA-targeted CTT1403. Mol Imaging Biol.

[69] Jiang Y, Liu Q, Wang G, Zhang J, Zhu Z, Chen X (2023). Evaluation of safety, biodistribution, and dosimetry of a long-acting radiolabeled somatostatin analog ^177^Lu-DOTA-EB-TATE with and without amino acid infusion. Clin Nucl Med.

[70] Iori M, Capponi PC, Rubagotti S, Esposizione LR, Seemann J, Pitzschler R (2017). Labelling of ^90^Y- and ^177^Lu-DOTA-bioconjugates for targeted radionuclide therapy: a comparison among manual, semiautomated, and fully automated synthesis. Contrast Media Mol Imaging.

[71] Reubi JC, Schar JC, Waser B, Wenger S, Heppeler A, Schmitt JS (2000). Affinity profiles for human somatostatin receptor subtypes SST1-SST5 of somatostatin radiotracers selected for scintigraphic and radiotherapeutic use. Eur J Nucl Med.

[72] Reubi JC, Waser B, Schaer JC, Laissue JA (2001). Somatostatin receptor sst1-sst5 expression in normal and neoplastic human tissues using receptor autoradiography with subtype-selective ligands. Eur J Nucl Med.

[73] Fani M, Braun F, Waser B, Beetschen K, Cescato R, Erchegyi J (2012). Unexpected sensitivity of sst2 antagonists to N-terminal radiometal modifications. J Nucl Med.

[74] Grant M, Kumar U (2010). The role of G-proteins in the dimerisation of human somatostatin receptor types 2 and 5. Regul Pept.

[75] Rocheville M, Lange DC, Kumar U, Sasi R, Patel RC, Patel YC (2000). Subtypes of the somatostatin receptor assemble as functional homo- and heterodimers. Journal of Biological Chemistry.

[76] Robertson MJ, Meyerowitz JG, Panova O, Borrelli K, Skiniotis G (2022). Plasticity in ligand recognition at somatostatin receptors. Nat Struct Mol Biol.

[77] Antunes P, Ginj M, Zhang H, Waser B, Baum RP, Reubi JC (2007). Are radiogallium-labelled DOTA-conjugated somatostatin analogues superior to those labelled with other radiometals?. Eur J Nucl Med Mol Imaging.

[78] Doctor A, Seifert V, Ullrich M, Hauser S, Pietzsch J (2020). Three-dimensional cell culture systems in radiopharmaceutical cancer research. Cancers (Basel).

[79] Seifert V, Richter S, Bechmann N, Bachmann M, Ziegler CG, Pietzsch J (2021). HIF2alpha-associated pseudohypoxia promotes radioresistance in pheochromocytoma: insights from 3D models. Cancers (Basel).

[80] Seifert V, Liers J, Kniess T, Richter S, Bechmann N, Feldmann A (2019). Fluorescent mouse pheochromocytoma spheroids expressing hypoxia-inducible factor 2 alpha: Morphologic and radiopharmacologic characterization. J Cell Biotechnol.

[81] Gerdekoohi SK, Vosoughi N, Tanha K, Assadi M, Ghafarian P, Rahmim A (2017). Implementation of absolute quantification in small-animal SPECT imaging: Phantom and animal studies. J Appl Clin Med Phys.

[82] Gupta A, Lee MS, Kim JH, Lee DS, Lee JS (2020). Preclinical voxel-based dosimetry in theranostics: a review. Nucl Med Mol Imaging.

[83] Hofheinz F, Dittrich S, Potzsch C, Hoff J (2010). Effects of cold sphere walls in PET phantom measurements on the volume reproducing threshold. Phys Med Biol.

[84] Sagisaka Y, Takahashi Y, Hosokawa S, Kanazawa N, Yamamoto H, Takai G (2024). Acquisition conditions for Lu-177 DOTATATE tmaging. Radiation.

[85] Schmitt A, Bernhardt P, Nilsson O, Ahlman H, Kolby L, Schmitt J (2003). Biodistribution and dosimetry of ^177^Lu-labeled [DOTA^0^,Tyr^3^]octreotate in male nude mice with human small cell lung cancer. Cancer Biother Radiopharm.

[86] Jahn U, Ilan E, Sandstrom M, Garske-Roman U, Lubberink M, Sundin A (2020). ^177^Lu-DOTATATE peptide receptor radionuclide therapy: dose response in small intestinal neuroendocrine tumors. Neuroendocrinology.

[87] Kamaldeep Loharkar S, Das T Basu S, Banerjee S (2022). Estimation of absorbed doses of indigenously produced "direct-route" lutetium-177-[^177^Lu]Lu-DOTA-TATE PRRT in normal organs and tumor lesions in patients of metastatic neuroendocrine tumors: comparison with no-carrier-added [^177^Lu]Lu-DOTA-TATE and the trend with multiple cycles. Cancer Biother Radiopharm.

