# Supplementary material for: The heterobivalent (SSTR2/albumin) radioligand [67Cu]Cu-NODAGA-cLAB4-TATE enables efficient somatostatin receptor radionuclide theranostics
Source: Theranostics. 2024 Aug 26;14(14):5371–87. doi: 10.7150/thno.100091 (PMC11413788; doi:10.7150/thno.100091)
Supplement: Supplementary file 1 — Supplementary materials and methods, figures and tables. [file thnov14p5371s1.pdf]

The heterobivalent (SSTR2/albumin) radioligand [^{67}Cu]Cu-NODAGA-cLAB4-TATE enables efficient somatostatin receptor radionuclide theranostics

- Supporting information -

Martin Ullrich[1*], Robert Wodtke[1], Florian Brandt[2], Robert Freudenberg[2], Jörg Kotzerke[2]; Susan Richter[3], Klaus Kopka[1,4,5,6], and Jens Pietzsch[1,4]

1. Helmholtz-Zentrum Dresden-Rossendorf, Institute of Radiopharmaceutical Cancer Research, Dresden, Germany
2. University Hospital Carl Gustav Carus at the Technische Universität Dresden, Klinik und Poliklinik für Nuklearmedizin, Dresden, Germany
3. University Hospital Carl Gustav Carus at the Technische Universität Dresden, Institute of Clinical Chemistry and Laboratory Medicine, Dresden, Germany
4. Technische Universität Dresden, School of Science, Faculty of Chemistry and Food Chemistry, Dresden, Germany
5. German Cancer Consortium (DKTK), Partner Site Dresden, Dresden, Germany
6. National Center for Tumor Diseases (NCT), Partner Site Dresden, University Cancer Center (UCC), Dresden, Germany

* Corresponding author and person to whom reprint requests should be addressed:

Dr. Martin Ullrich

Helmholtz-Zentrum Dresden-Rossendorf

Institute of Radiopharmaceutical Cancer Research

Bautzner Landstraße 400, 01328 Dresden, Germany

Phone: +49-351-2604046, Fax: +49-351-26012622

E-mail: m.ullrich@hzdr.de

Table of Contents

I. Radiolabeling of NODAGA-TATE variants with copper-67 3

Radiolabeling efficiency and peptide contents within the radioligand preparations 3

II. Animal cohorts and treatments 3

Method: Cohort assembly and allocation to treatments 3

III. Binding constants of gallium-68-, lutetium-177-, copper-64-, and copper-67-labeled TATE variants in intact cells and cell homogenates 4

Binding constants measured with intact MPC cells and cell homogenates 4

Hypotheses on lower binding capacities of MPC cells for copper-64- and copper-67-labeled **NODAGA-TATE** variants compared to gallium-68- and lutetium-177-labeled versions of **DOTA-TATE** 4

IV. Inhibitory constants of metal-free, lutetium-, and copper-labeled TATE variants 6

Method: Competition binding of SSTR2 ligands against **[^{64}Cu]Cu-DOTA-TATE** in MPC cell homogenates 7

Inhibitory constants of metal-free and metalated **DOTA-TATE** and **NODAGA-TATE** variants 7

V. Treatment effects of lutetium-177- and copper-67-labeled TATE variants on MPC tumor spheroids 8

Method: Cultivation and treatment of tumor spheroids 8

Method: Radioligand uptake in tumor spheroids 9

Method: Analysis of spheroid growth 9

Uptake of copper-67-labeled **NODAGA-TATE** variants and growth inhibition in MPC tumor spheroids 9

VI. SPECT imaging: acquisition, reconstruction, resolution, and activity calibration 12

Method: Image acquisition and reconstruction 13

Method: Spatial resolution measurements and activity calibration 13

Count rates, spatial resolution, and quantification accuracy in SPECT imaging of lutetium-177 and copper-67 14

VII. SPECT imaging of lutetium-177- and copper-67-labeled TATE variants in tumor-bearing mice 16

Image series from treatments with radioligands at a molar activity of 40 MBq/nmol 16

Image series from treatments with radioligands at a molar activity of 20 MBq/nmol 17

Method: Extraction of tissue-specific activity concentrations from SPECT/CT images 18

VIII. Pharmacokinetics of lutetium-177- and copper-67-labeled TATE variants in MPC allograft mice 20

Tissue-specific pharmacokinetic profiles in mouse organs and MPC tumors 20

Subcutaneous MPC tumor allografts are characterized by a rapid release of SSTR2 radioligands. 22

IX. Projected human pharmacokinetics and dose estimations for treatments with lutetium-177- and copper-67-labeled TATE variants 23

Projected time-activity curves in humans extrapolated from pharmacokinetic profiles in mice 23

Projected human organ doses for radioligand treatments at a molar activity of 40 MBq/nmol 24

X. Treatment effects of lutetium-177- and copper-67-labeled TATE variants in MPC allograft mice 25

Growth-reducing effects on subcutaneous MPC tumor allografts 25

Absorbed doses in MPC tumors 26

Time courses in body weight of MPC allograft mice treated with lutetium-177- and copper-67-labeled TATE variants 26

I. Radiolabeling of NODAGA-TATE variants with copper-67

Radiolabeling efficiency and peptide contents within the radioligand preparations

Radiolabeling of **NODAGA-TATE** variants in sodium ascorbate buffer enables straightforward and efficient complexation of copper-67 even under mild conditions (40°C). Without additional purification, the achieved radiochemical purities of > 96 % are comparable to what is achieved in routine radiolabeling procedures for **[^{177}Lu]Lu-DOTA-TATE** at 80°C. This confirms that, due to its high molar activity and chemical purity, photonuclear reaction-produced [^{67}Cu]$CuCl_2$ is well suited for radiolabeling of NODAGA-functionalized peptides. Notably, the remaining non-labeled precursor fraction was explicitly not saturated with the corresponding non-radioactive metal ions (in natural isotope composition) to match typical formulations of most clinical SSTR2 radioligand preparations [37, 70]. Therefore, the ratios between the distinct versions of the SSTR2 ligand within the radioligand preparation (radiometal complex, non-radioactive metal complexes, metal-free precursor) can be expected to modulate the pharmacokinetics to a certain extent.

II. Animal cohorts and treatments

Method: Cohort assembly and allocation to treatments

Mice with tumors of 6 ± 2 mm in diameter were grouped according to a similar average tumor volume per treatment group. The allocation to therapy and control groups was random. Consecutive experiments were performed according to the identical treatment protocol (Table S 1).

Table S 1: Animal cohorts for treatment with lutetium-177- and copper-67-labeled TATE variants

**Cohort**	**TATE variant**	**RN**	A_m	**ID** $_{activity}$ ^{a}	**ID** $_{molar}$ ^{b}	**BW**	**TV**	**Animals**
#			[MBq/nmol]	[MBq]	[nmol]	[g]	[mm^3]	*n* (per exp.)
1	Control/vehicle		–	–	–	30.0 ± 0.79	84.7 ± 4.72	8 (3, 3, 2)
2	DOTA	^{177}Lu	39.6 ± 0.09	50.4 ± 0.44	1.27	30.3 ± 0.37	95.2 ± 12.2	5 (5)
3			22.6 ± 1.42	48.3 ± 1.67	2.14	31.3 ± 0.55	81.9 ± 7.75	6 (3, 3)
4	NODAGA	^{67}Cu	40.3 ± 0.76	50.7 ± 0.48	1.26	29.1 ± 0.89	77.9 ± 7.39	6 (4, 2)
5			24.6 ± 0.67	47.7 ± 1.74	1.94	31.9 ± 0.66	82.7 ± 12.9	5 (3, 2)
6	NODAGA-cLAB4	^{67}Cu	41.5 ± 0.56	51.1 ± 1.40	1.23	29.9 v0.79	78.1 ± 11.5	6 (3, 3)
7			21.5 ± 0.54	51.7 ± 2.10	2.40	30.2 ± 0.77	91.2 ± 21.9	5 (2, 1, 2)

(A_m) molar activity at treatment start; (BW) body weight at treatment start; (ID) initial doses referring to the amount of ^{a} activity or to the ^{b} equivalent molar amount of radioligand administered at treatment start, respectively; (RN) radionuclide; (TV) tumor volume at treatment start; (*n*) number of animals per group and per independent experiment; data presented as means ± standard error

III. Binding constants of gallium-68-, lutetium-177-, copper-64-, and copper-67-labeled TATE variants in intact cells and cell homogenates

The development of novel SSTR2 ligands entails the characterization of their binding constants, which is often performed with radioligands in a saturation or competition binding assay format. Since SSTR2 is a G-protein-coupled transmembrane protein, receptor samples for binding assays are typically prepared from tissue sections, cell membranes, cell homogenates, or intact cells [34, 71-73]. In the present study, binding constants measured in a microplate assay with intact MPC cells were compared with those measured in an MPC cell homogenate assay to examine whether functional cellular responses (e.g. receptor-mediated endocytosis, endosomal/lysosomal receptor traffic) may influence the results.

Binding constants measured with intact MPC cells and cell homogenates

The trends in K_d values and B_{max} values within the series of gallium-68-, lutetium-177-, copper-64-, and copper-67-labeled-labled TATE variants were largely consistent between the two binding assays using intact MPC cells or cell homogenates (Figure S 1). However, B_{max} values measured with the intact cell assay tend to be higher compared to the homogenate assay. This could suggest that, in intact cells, receptor-mediated endocytosis followed by endosomal recycling provides recurrent availability of SSTR2 on the cell surface without the concomitant stoichiometric release of radioligand, enabling repeated ligand binding by the same receptor molecule. Therefore, B_{max} values measured for different radioligands in intact MPC cells not only depend on the number of SSTR binding sites but also on their uptake and recycling rates. Further general aspects to be considered in binding assays with intact cells or cell homogenates have been discussed elsewhere [34].

Hypotheses on lower binding capacities of MPC cells for copper-64- and copper-67-labeled ***NODAGA-TATE*** *variants compared to gallium-68- and lutetium-177-labeled versions of* ***DOTA-TATE***

In binding studies performed with intact cells, radioligand-specific uptake ratios as well as rate constants for intracellular SSTR2 traffic (lysosomal trapping *versus* endosomal recycling) have been reported to functionally affect the measurement of B_{max} values *in vitro* [45, 46]. For example, intact cells exhibit higher binding capacities for the SSTR2 antagonist **[^{177}Lu]Lu-DOTA-JR11** compared to the agonist **[^{177}Lu]Lu-DOTA-TATE**, but not membrane preparations [44]. However, radioligand-specific trends in binding capacities are reproducibly measured across a broad range of SSTR2 source material including intact MPC cells, cell homogenates, and tumor spheroids, thus excluding functional interference with the B_{max} measurements in the present study.

Both copper-NODAGA and copper-DOTA complexes exhibit a single negative net charge compared to the neutral lutetium-DOTA complex [41]. Therefore, hypotheses on radioligand-specific binding capacities may rather consider electrostatic effects on the interactions between the radioligands with SSTR2 and/or the cell surface with possible consequences for binding/re-binding equilibrium. A radioligand-specific selectivity for different receptor conformations may also exist. For example, SSTR2 conformations have been reported to change upon dimerization or coupling/uncoupling of G-proteins [74-76]. G-protein-coupled and non-coupled receptors may also reside in distinct membrane microenvironments and their existence may vary in different cellular origins, e.g., recombinant, and natural cells [42]. In MPC cell lysates, antibodies recognize SSTR2 at a molecular weight between 85 and 100 kDa which is approximately double the weight of the monomer [33]. Thus, the possibility arises that the natural existence of the SSTR2 dimer may have a role in the net charge-related differences in binding capacities between radiocopper-labeled **DOTA-/NODAGA-TATE** variants and **[^{177}Lu]Lu-DOTA-TATE**.

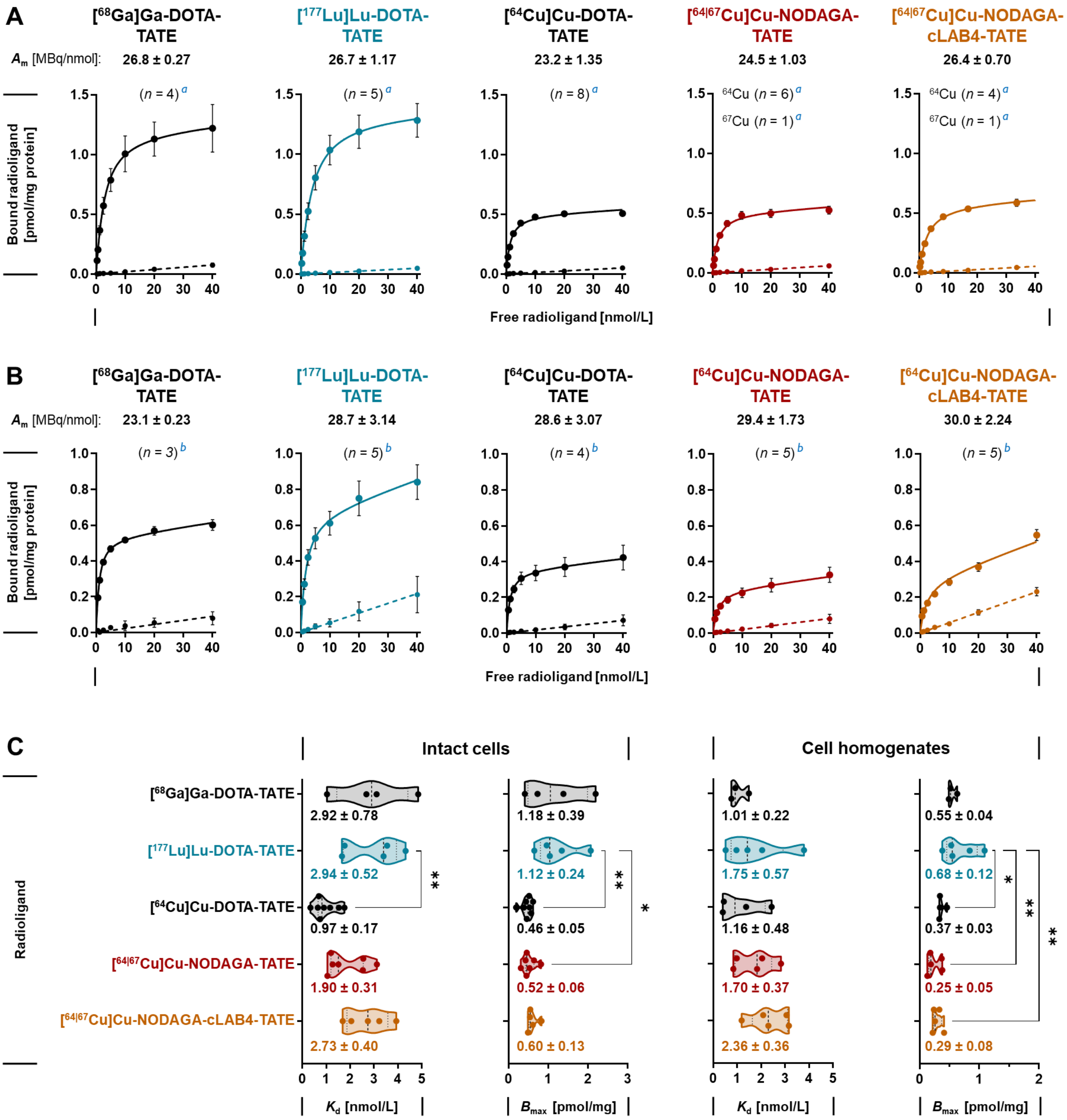

Figure S 1: Binding of SSTR2 gallium-68-, lutetium-177-, copper-64-, and copper-67-labeled labeled TATE variants in different MPC cell preparations; (A) Total and non-specific binding towards intact cell monolayer; (B) Total and non-specific binding towards cell homogenates; (continuous line) total binding; (dashed line) non-specific binding; (C) Comparison of binding constants measured with intact cells and cell homogenates, respectively; binding was measured at free radioligand concentrations between 0.3–40 nmol/L; non-specific binding was assessed in presence of 1 nmol/L acetyl-TATE; (A_m) molar activity at incubation start; number of independent experiments (n) each performed in [a] triplicate or [b] duplicate; data presented as means ± standard error; significance of difference compared to **[^{177}Lu]DOTA-TATE**: * $p < 0.05$, ** $p < 0.01$

IV. Inhibitory constants of metal-free, lutetium-, and copper-labeled TATE variants

Small structural modifications, chelator substitution or metal replacement have been reported to considerably affect the binding affinity of SSTR2 targeting peptide-chelator conjugates including agonists

and antagonist [71, 73, 77]. As shown in these studies, the effects on binding affinity resulting from changes in the metal-chelate complex can vary considerably between conjugate types. In the present study, it can be assumed that different ligand versions in the preparations of **[^{67}Cu]Cu-NODAGA-TATE**, **[^{67}Cu]Cu-NODAGA-cLAB4-TATE** and **[^{177}Lu]Lu-DOTA-TATE** (radiometal complex, non-radioactive metal complexes, metal-free precursor) modulate their pharmacological behavior to a certain extent. Therefore, comparing the inhibitory constants of the corresponding non-radioactive reference ligands (metal-free and metalated versions) may help to estimate their mutual interference *in vitro* and *in vivo*.

*Method: Competition binding of SSTR2 ligands against **[^{64}Cu]Cu-DOTA-TATE** in MPC cell homogenates*

Cryo-preserved MPC cells (1×10^{7}/mL) were resuspended in ice-cold RPMI 1640 medium supplemented with cOmplete™ EDTA-free proteinase inhibitor (Roche, Basel Switzerland) and homogenized using a Dounce homogenizer. A series of homogenates, each from 1×10^{6} cells, were incubated with 2 nmol/L **[^{64}Cu]Cu-DOTA-TATE** (A_m = 43.9 MBq/nmol) in presence of additional competitor at increasing concentrations between 10 pmol/L and 5 µmol/L in a final volume of 0.5 mL for 1 hour at 37°C. Incubation was stopped by soaking the cell homogenates into Whatman GF/C collection filters (GE Healthcare, Chicago, IL, USA; presoaked in 0.3 % (v/v) polyethyleneimine for 90 min). Filters were washed with ice-cold Dulbecco's phosphate-buffered saline using a harvester (Brandel, Gaithersburg, MD, USA). Filter-bound activity was measured using the γ counter Wizard (PerkinElmer), corrected for plastic-bound background, and normalized to bound activity without competitor. Inhibitory constants were calculated by non-linear regression analysis using the 'One-site Fit K_i' model implemented in Prism 10 with binding parameters for **[^{64}Cu]Cu-DOTA-TATE** constrained to K_d = 1.16 nmol/L and c = 2 nmol/L.

*Inhibitory constants of metal-free and metalated **DOTA-TATE** and **NODAGA-TATE** variants*

In the present study, competition binding against **[^{64}Cu]Cu-DOTA-TATE** showed similar inhibitory constants (K_i values between 0.76 and 1.78 nmol/L) for the non-radioactive metalated conjugates **[^{nat}Cu]Cu-NODAGA-TATE**, **[^{nat}Cu]Cu-NODAGA-cLAB4-TATE** and **[^{nat}Lu]Lu-DOTA-TATE** as well as for their corresponding metal-free precursors (Figure S 2). This indicates that the incorporated metal and the net charge of the metal complex have no influence on the binding of the different ligands versions towards SSTR2. This further suggests that the content of non-radioactive precursor in the radioligand preparations had no influence on affinity measurement in the present study.

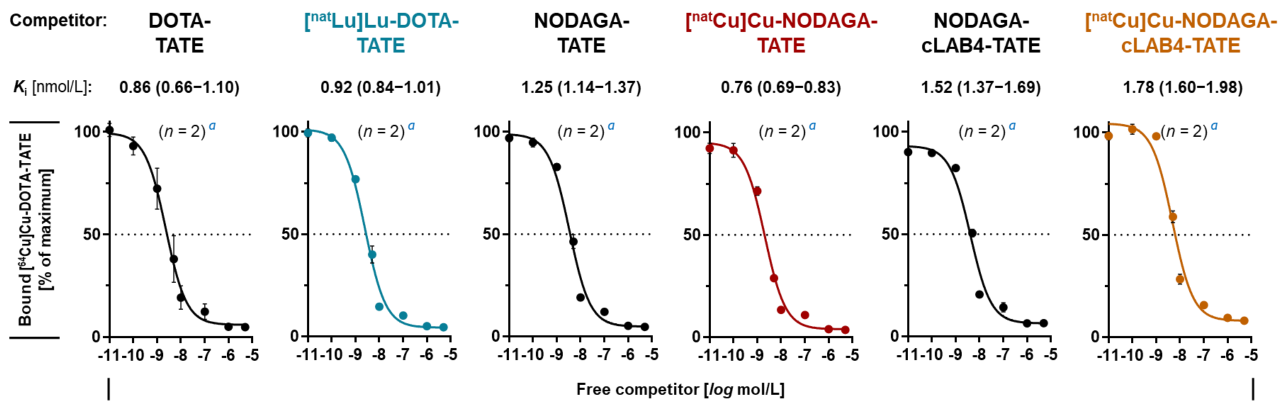

Figure S 2: Competitive binding of metal-free, lutetium, and copper-labeled TATE variants against **[⁶⁴Cu]Cu-DOTA-TATE** in MPC cell homogenates; competitors were investigated either metal-free or 'saturated' with indicated metal ions in naturally occurring isotope composition; (K_i) inhibition constants against **[⁶⁴Cu]Cu-DOTA-TATE** (K_d = 1.16 nmol/L, A_m = 43.9 MBq/nmol) at a molar concentration of 2 nmol/L; number of independent experiments (*n*) each performed in [a] triplicate; data presented as means with a confidence interval of 68 %

V. Treatment effects of lutetium-177- and copper-67-labeled TATE variants on MPC tumor spheroids

As three-dimensional models, tumor spheroids offer the possibility to study the response to targeted radionuclide therapies, taking into account tissue-related characteristics which are absent in monolayer cultures such as restricted perfusion, hypoxic regions, genetic and phenotypic heterogeneity, gradients and barriers in nutrient supply as well as cell-cell and cell-matrix communications [78, 79]. In this study, an MPC tumor spheroid model was investigated to determine the initial concentrations of copper-67-labeled **NODAGA-TATE** variants enabling adequate uptake and growth inhibition *in vitro*.

Method: Cultivation and treatment of tumor spheroids

Tumor spheroids were generated from an initial number of 1000 MPC cells seeded into concave-bottom ultra-low attachment plates (Perkin Elmer Life Sciences, Waltham, MA, USA) and further cultured using the liquid-overlay technique as described previously [80]. When reaching a diameter of 450–550 µm, spheroids were treated with radioligand added to the culture medium at increasing initial activity concentrations of 20–400 kBq/mL in a final volume of 0.2 mL. Depending on the molar activities of the radioligand preparations at 20 or 40 MBq/nmol, the initial activity concentration range corresponded to molar concentration ranges of 2–40 or 1–20 nmol/L, respectively. After 2 hours of radioligand exposure at 37° C, radioactive medium was partially replaced three times with non-radioactive medium, each at a ratio of 1:3, to remove 96.3 % of the radioligand. During follow-up, medium was partially replaced every 2–4 days with fresh medium at a ratio of 1:2.

Method: Radioligand uptake in tumor spheroids

Spheroids were treated with radioligands as described above. Total uptake of radioligands was determined for exposure at the highest initial activity concentration of 400 kBq/mL. Non-specific binding was determined in presence of 1 µmol acetyl-TATE. Binding to plastic surfaces was determined in spheroid-free cavities. Spheroids were soaked into UniFilter-96 GF/C microplates (PerkinElmer) and washed three times with Dulbecco's phosphate-buffered saline containing 0.9 mmol/L $CaCl_2$ and 0.5 mmol/L $MgCl_2$ using the UniFilter-96 Cell harvester (PerkinElmer). Activity of filters was measured using the gamma counter Wizard² 2480 (PerkinElmer). Activity standards were prepared from serially diluted incubation media. Specific binding was calculated as percent initial dose normalized to spheroid volume (% ID/mm^3).

Method: Analysis of spheroid growth

Spheroid size was monitored and documented every 2–4 days after treatment start for up to 35 days using the microscope Axiovert 40 CFL (Carl Zeiss, Oberkochen, Germany). Bright field images were converted into ECAT files. Spheroids were identified and two-dimensional ROIs were drawn using a semi-automated contrast-based delineation workflow in Rover (ABX, Radeberg, Germany) with contrast thresholds between 1 and 180. Delineations were manually reviewed and adjusted in case of unfavorable contrast conditions, for example due to increased amounts of debris. Spheroids circular areas were extracted, and changes were analyzed in 32 spheroids per treatment condition. Fractions of controlled spheroids were determined for indicated time points. Progression-free control (PFC) was defined as < 1.2-fold change in spheroid circular area, overall control (OC) was defined as < 2.2-fold change in spheroid circular area. Median PFC and OC were calculated using the survival analysis tool implemented in Prism 10 (GraphPad Software). For calculation of half-maximal spheroid control doses (SCD50) at specific time points, controlled fractions were plotted against a series of increasing radioligand concentrations and analyzed by non-linear regression using the sigmoidal '[Agonist] vs. response – Variable slope (four parameters)' model implemented in Prism 10 (GraphPad Software). The 'Top' and 'Bottom' parameters were constrained to 1 and 0, respectively. The 'Hillslope' parameter was constrained to the best-fit value for **[^{177}Lu]Lu-DOTA-TATE** treatment.

Uptake of copper-67-labeled ***NODAGA-TATE*** *variants and growth inhibition in MPC tumor spheroids*

The activity concentration initially required for the copper-67-labeled **NODAGA-TATE** variants to provide adequate uptake and treatment efficacy was determined from growth inhibition of MPC tumor

spheroids and compared to **[^{177}Lu]Lu-DOTA-TATE** (Figure S 3 A). Upon treatment with the highest tested activity concentration (400 kBq/mL), the effective uptake of the copper-67-labeled **NODAGA-TATE** variants in spheroids was 72 % or 49% lower compared to **[^{177}Lu]Lu-DOTA-TATE**, depending on their molar activity of 40 or 20 MBq/nmol, respectively (Figure S 3 B, Table S 2). Due to lower effective uptake, faster release, and shorter physical half-life of the radionuclide, the copper-67-labeled **NODAGA-TATE** variants required a four- to six-fold higher initial activity concentration to provide equivalent control of spheroid growth to **[^{177}Lu]Lu-DOTA-TATE** *in vitro* (Figure S 3 C–D, Table S 2). Further details on the analysis of growth-reducing effects on spheroids are provided in Figure S 4 A–C.

This outcome is consistent with the lower binding capacities and faster release rates measured for the copper-64-labeled versions of the radioligands. Of note, the initial concentrations of copper-67-labeled **NODAGA-TATE** variants that provided half-maximal control of MPC spheroid growth are within the same range as has been recently reported for growth inhibition of genetically modified QGP1-SSTR2+ spheroids after treatment with the albumin-affine **[^{67}Cu]Cu-DOTA-EB-TATE** conjugate (200–1,000 kBq/mL) [50]. The present results indicate that effective treatment with the copper-67-labeled **NODAGA-TATE** variants requires high initial radioligand concentrations and sufficiently long radioligand exposure *in vivo* to maximize effective initial uptake and to compensate the faster release from tumor cells.

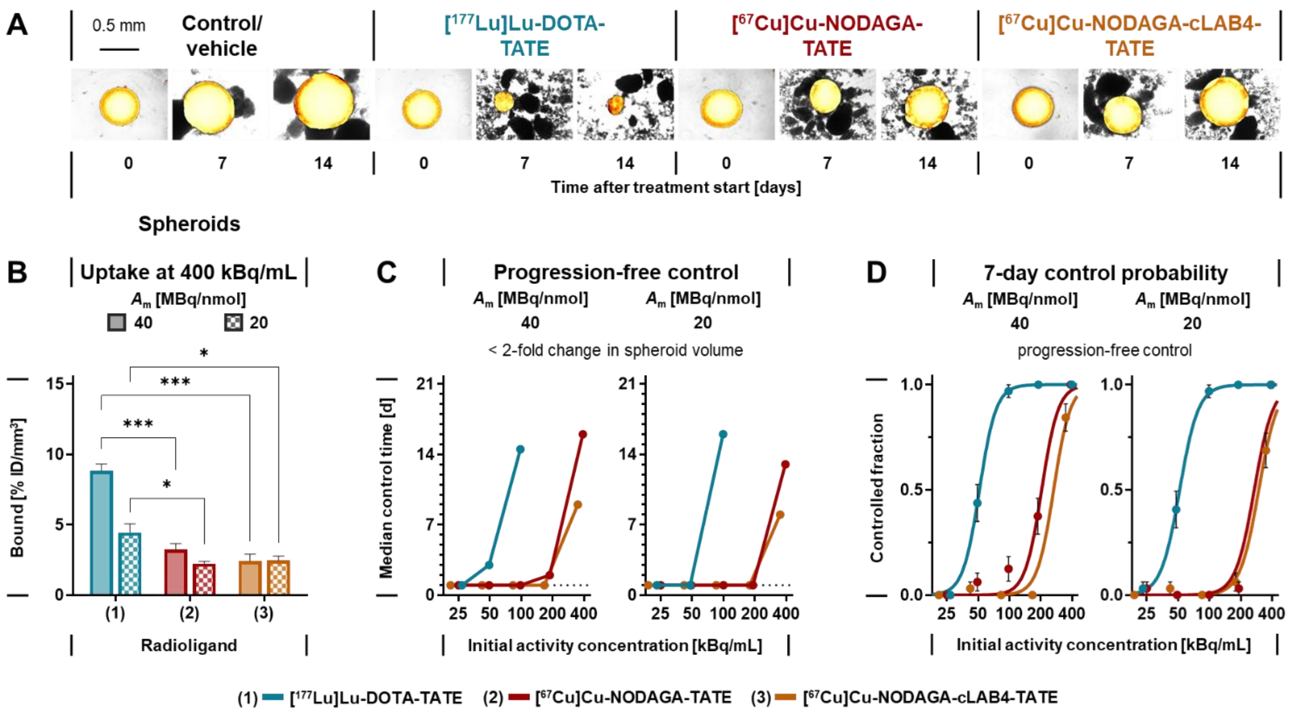

Figure S 3: Uptake and growth-inhibitory effects of lutetium-177- and copper-67-labeled TATE variants in MPC tumor spheroids; initial activity concentrations (IAC) 20−400 kBq/mL and initial activity doses (ID) 4−80 kBq/per microwell followed by radioligand removal; IAC corresponded to molar concentrations between 2−40 nmol/L (A_m = 20 MBq/nmol) or 1−20 nmol/L (A_m = 40 MBq/nmol); (A) Microscopic images of spheroid growth after radioligand treatment; in yellow: regions of interest to determine spheroid circular areas; (B) Specific binding and uptake in spheroids after treatment with radioligands at highest IAC; (C) Median progression-free control after treatment with radioligands at increasing IAC; (D) Dependence of progression-free control on the IAC of the radioligands 7 days after treatment start; progression-free control is defined as < 1.2-fold change in spheroid circular area; dotted lines indicate base-line values measured in spheroids without treatment; (A_m) molar activity at treatment start; data presented as means ± standard error; significance of differences: * $p < 0.05$, ** $p < 0.01$, *** $p < 0.001$

Table S 2: Uptake of lutetium-177- and copper-67-labeled TATE variants in MPC tumor spheroids and inhibition of spheroid growth

TATE variant	RN	A_m [MBq/nmol]	IAC$_{max}$ [kBq/mL]	Uptake$_{max}$ [% ID/mm³]	PFC [days]	OC [days]	SCD50$_{7d\ PFC}$ [a] IAC [kBq/mL]	SCD50$_{14d\ OC}$ [b] IAC [kBq/mL]
Control/vehicle	–	–	–	–	1	8	–	–
DOTA	^{177}Lu	41.1	395	8.8 ± 0.5	> 35	> 35	51.5 ± 0.50	44.0 ± 6.14
		20.6	388	4.4 ± 0.6	> 35	> 35	51.8 ± 0.31	47.8 ± 3.44
NODAGA	^{67}Cu	43.6	388	3.2 ± 0.4	16	23	204 ± 8.89	204 ± 29.9
		20.6	391	2.2 ± 0.2	13	16	269 ± 26.9	297 ± 24.8
NODAGA-cLAB4	^{67}Cu	42.3	347	2.4 ± 0.5	9	16	265 ± 11.1	360 ± 57.5
		27.5	348	2.5 ± 0.3	8	16	297 ± 4.19	339 ± 24.5

(A_m) molar activity at treatment start, (IAC) initial activity concentrations between 20–400 kBq/mL corresponding to initial doses (ID) between 4−80 kBq/per microwell followed by radioligand removal; (OC) overall control defined as < 2.2-fold change in spheroid circular area and (PFC) progression-free control defined as < 1.2-fold change in spheroid circular area compared to treatment start, respectively; (RN) radionuclide; (SCD50) half-maximal spheroid control doses are defined as the IAC providing [a] progression-free control for 7 days or [b] overall control for 14 days, in 50 % of spheroids; 32 spheroids were monitored for each treatment condition, uptake and SCD50 are presented as means ± standard error, PFC and OC are presented as medians

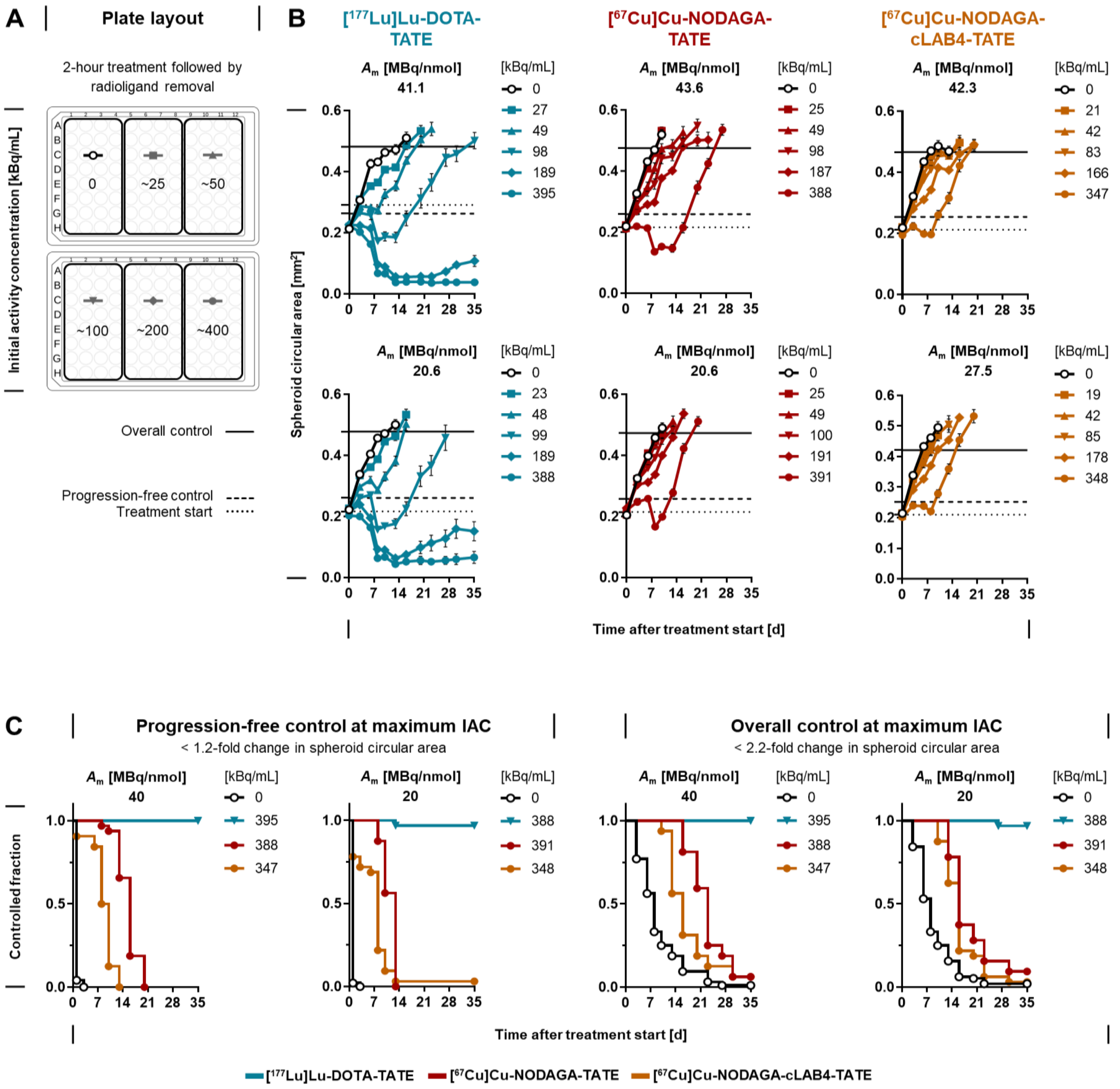

Figure S 4: Growth-inhibitory effects of lutetium-177- and copper-67-labeled TATE variants on MPC tumor spheroids; initial activity concentrations (IAC) 20–400 kBq/mL; initial activity doses (ID) 4–80 kBq/per microwell followed by radioligand removal from the culture medium; IAC corresponded to molar concentrations between 1–20 nmol/L (A_m = 40 MBq/nmol) or 2–40 nmol/L (A_m = 20 MBq/nmol); (A) Plate layout for exposure of spheroid arrays to increasing IAC of the radioligands; data presented as means ± standard error; (B) Changes in spheroid volume in response to treatments at increasing IAC; measured IAC in the incubation medium; horizontal lines indicate specific thresholds for survival analyses; (C) Treatment effects of the highest tested IAC on progression-free and overall control; progression-free control is defined as < 1.2-fold change in spheroid circular area compared to treatment start and overall control is defined as < 2.2-fold change in spheroid circular area

VI. SPECT imaging: acquisition, reconstruction, resolution, and activity calibration

Absolute quantification in preclinical single-photon emission computed tomography (SPECT) imaging provides a valuable non-invasive alternative to terminal biodistribution studies for monitoring the activity profiles of photon-emitting radioactive probes in small laboratory animals providing the basis for drug development, organ dosimetry, and therapy response assessment [81]. Hence, the quantitative

accuracy in SPECT imaging is essential for reliably assessing tumor uptake and tumor-to-normal tissue ratios necessary to perform dosimetry [82]. Accurate image quantitation in small animal SPECT is always challenging due to the limitations in the instrumentation and imaging process; therefore, it is important to evaluate performance parameters of preclinical SPECT systems used for theranostic radionuclides during dosimetry studies [82]. In the present study, SPECT images of lutetium-177- and copper-67-loaded activity phantoms were analyzed to evaluate radionuclide-specific count rates, spatial resolution, and quantifications accuracy.

Method: Image acquisition and reconstruction

Small animal single-photon emission computed tomography was performed using the nanoScan® SPECT/CT (Mediso Medical Imaging Systems, Budapest, Hungary) equipped with the APT56 aperture consisting of four M^3 multi-pinhole collimators providing a 30×30 mm axial field of view (FOV). Photon emission was recorded using frame times between 60 and 240 s (scan times between 20 and 80 min) and binned within the 20 % energy windows of the radionuclide-specific photopeaks for lutetium-177 (56 [X-ray], 113 , and 208 keV) and copper-67 (93 and 185 keV). With each SPECT scan, a corresponding CT image was recorded and used for anatomical referencing and attenuation correction. Images were reconstructed using the Tera-Tomo™ three-dimensional (3D) algorithm with high dynamic range, a voxel size of 0.23 mm, and corrections for scatter and attenuation.

Method: Spatial resolution measurements and activity calibration

Five-mL syringes filled with 2 mL of 0.154 mol/L NaCl(aq) containing 15 MBq of $[^{177}Lu]LuCl_3$ or $[^{67}Cu]CuCl_2$ served as resolution and activity phantoms. Spatial resolution of SPECT images was determined using the 'Resolution tool' implemented in Rover version 3.0.77h (ABX) as described elsewhere [83], with some modifications. In brief, a 20 mm square region-of-interest (ROI) was centered over the syringe contents and split into a series of ten axially stacked sub-ROIs. For each sub-ROI, the effective spatial resolution was determined from its radial voxel intensity profile by calculating the full width at half-maximum (FWHM) of the point spread function (PSF). The FWHM values of all sub-ROIs along the axial length of the syringe contents were averaged and expressed in mm. The activity values of voxel intensities in SPECT images were calibrated by determining the conversion factor between the activity of the syringe contents measured in an ISOMED 2010 dose calibrator (NUVIA Instruments, Dresden, Germany) and the corresponding total number of counts in a reconstructed SPECT image with a 50 mm axial FOV.

Count rates, spatial resolution, and quantification accuracy in SPECT imaging of lutetium-177 and copper-67

Within the radionuclide-specific energy windows of the photopeaks (Figure S 5 A), copper-67 provided a 4.2-fold higher total number of counts collected per second and MBq (166 cps/MBq) compared to lutetium-177 (39 cps/MBq). This indicates that small animal SPECT imaging of copper-67 has higher sensitivity compared to lutetium-177. This advantage would be even higher in clinical settings since image recording of lutetium-177 typically includes only the 112 and 208 keV photopeaks [84].

SPECT images of the lutetium-177 and copper-67 contents within the syringe phantoms showed comparable radial voxel intensity profiles with a slight edge-intensifying effect of approximately 20 % (Figure S 5 B–C). The effective spatial resolution was higher in SPECT images of copper-67 (0.83 mm) compared to lutetium-177 (0.95 mm) (Figure S 5 D). The voxels from the syringe contents showed a similar intensity distribution for both lutetium-177 and copper-67, providing equal accuracy in image quantification (Figure S 5 E–F).

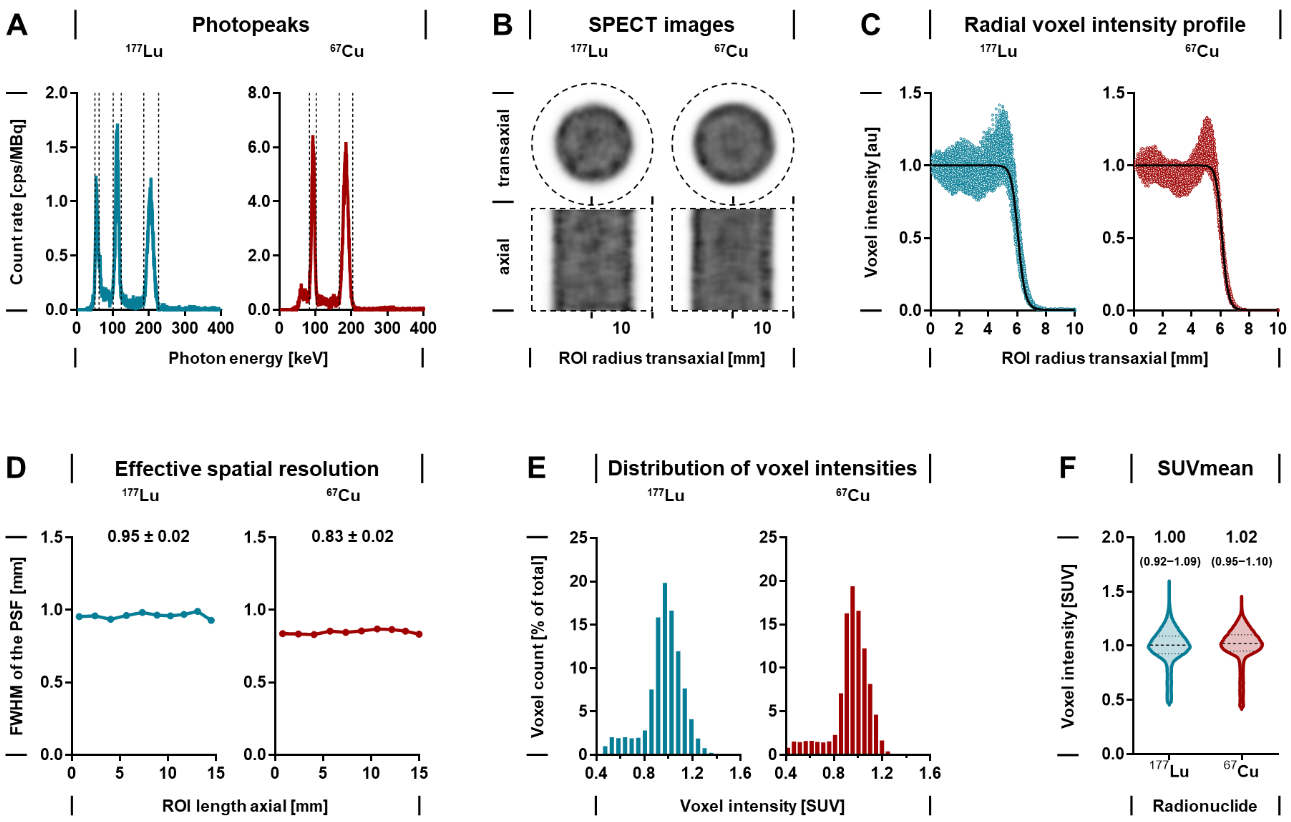

Figure S 5: Spatial resolution and quantification accuracy in SPECT imaging of lutetium-177and copper-67; 5-mL-syringe activity phantoms containing activity concentration of 7.5 MBq/mL were measured using the Mediso nanoScan® SPECT/CT equipped with the APT56 aperture consisting of four multi-pinhole collimators; (A) Energy spectra, photon count rates, and 20 % energy windows of the recorded photopeaks for imaging of lutetium-177 (56, 113, and 208 keV) and copper-67 (93 and 185 keV); (B) Planar SPECT images of activity phantoms; dashed fields indicate ROIs used for image analysis; (C) Radial voxel intensity profiles showing the decrease in contrast at the edges of the activity phantoms along the transaxial radius of the ROI; (D) Effective spatial resolution along the axial length of the analyzed ROI, spatial resolution was determined as full width at half-maximum (FWHM) of the point spread function (PSF), means ± standard error; (E) Distribution of voxel intensities within the activity phantoms; the analyzed ROI included all voxels above the minimum intensity threshold of 30 %; (F) Statistics of the mean standardized uptake value (SUVmean) measured in the activity phantoms; median with 25th and 75th percentiles

VII. SPECT imaging of lutetium-177- and copper-67-labeled TATE variants in tumor-bearing mice

Image series from treatments with radioligands at a molar activity of 40 MBq/nmol

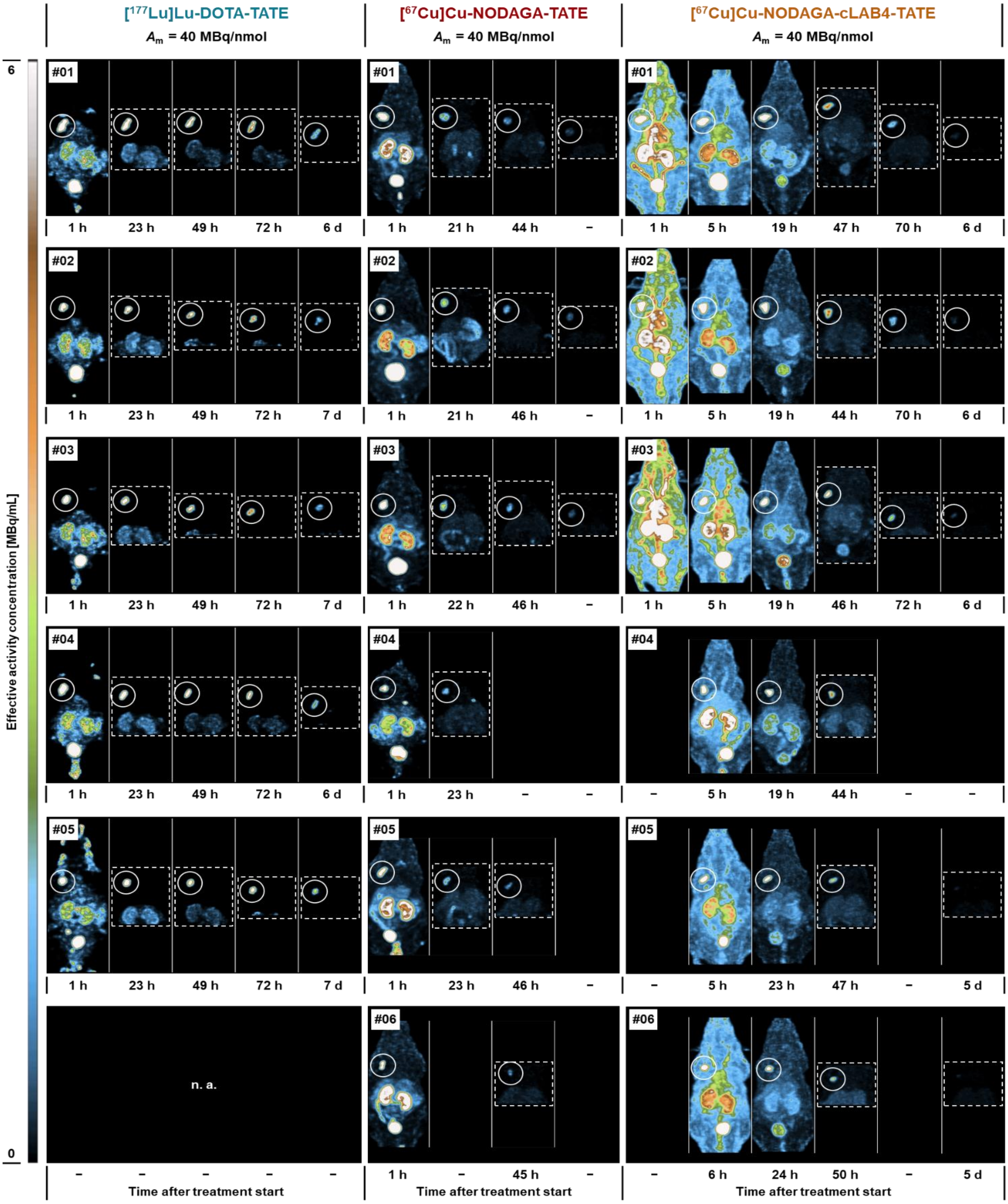

Figure S 6: SPECT image series of MPC allograft mice treated with lutetium-177- and copper-67-labeled TATE variants at a molar activity of 40 MBq/nmol; animals received an initial activity dose of 50 MBq corresponding to a molar amount of 1.25 nmol; maximum intensity projections; images presented without decay correction

Image series from treatments with radioligands at a molar activity of 20 MBq/nmol

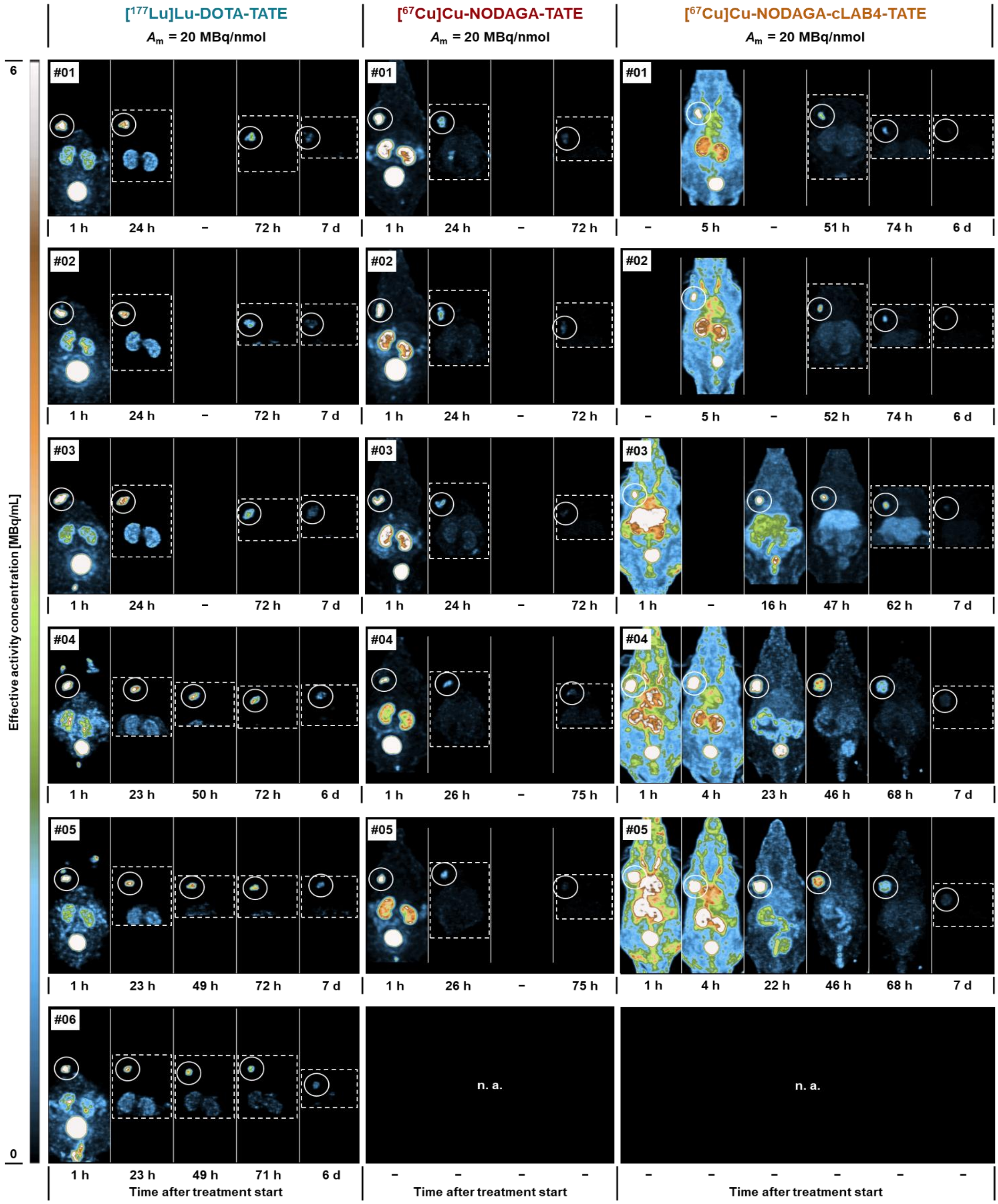

Figure S 7: SPECT image series of MPC allograft mice treated with lutetium-177- and copper-67-labeled TATE variants at a molar activity of 20 MBq/nmol; animals received an initial activity dose of 50 MBq corresponding to a molar amount of 2.5 nmol; maximum intensity projections; **[^{67}Cu]Cu-NODAGA-cLAB4-TATE** series: animal #03 was excluded from the analysis of liver and total body pharmacokinetics due to higher amounts (8 %) of residual [^{67}Cu]Cu^{2+} in the radioligand preparation resulting in increased liver retention, animal #04 was excluded form analysis of total body pharmacokinetics due to accidental oral incorporation of copper-67-contaminated bedding materials resulting in intestinal retention; images presented without decay correction

Method: Extraction of tissue-specific activity concentrations from SPECT/CT images

Tissue-specific regions of interest (ROIs) were generated in ROVER 3.0.77h (ABX). Sphere masks were positioned to pre-select tissue-specific regions for delineation. ROIs were generated within the masks using tissue-specific threshold-based delineation (% of maximum voxel intensity within the given mask) as follows: Heart contents (right atrium, > 5 %, Figure S 8 A); liver (right lobe region, > 5 %, Figure S 8 B); kidneys (full organ, > 40 %, medullary regions and renal pelvis manually included, Figure S 8 C), tumor (> 30 %, Figure S 8 D), total body (> 0.5 %, urinary bladder excluded, Figure S 8 E), urinary bladder (> 5 %, Figure S 8 F). For organ-specific dose estimations extracted activity concentrations were further converted into total organ activity per initial activity dose [MBq × h/MBq] using a realistic dosimetry model for 30-g-mice [35] implemented in Olinda 2.2.3 (Hermes Medical Solutions AB).

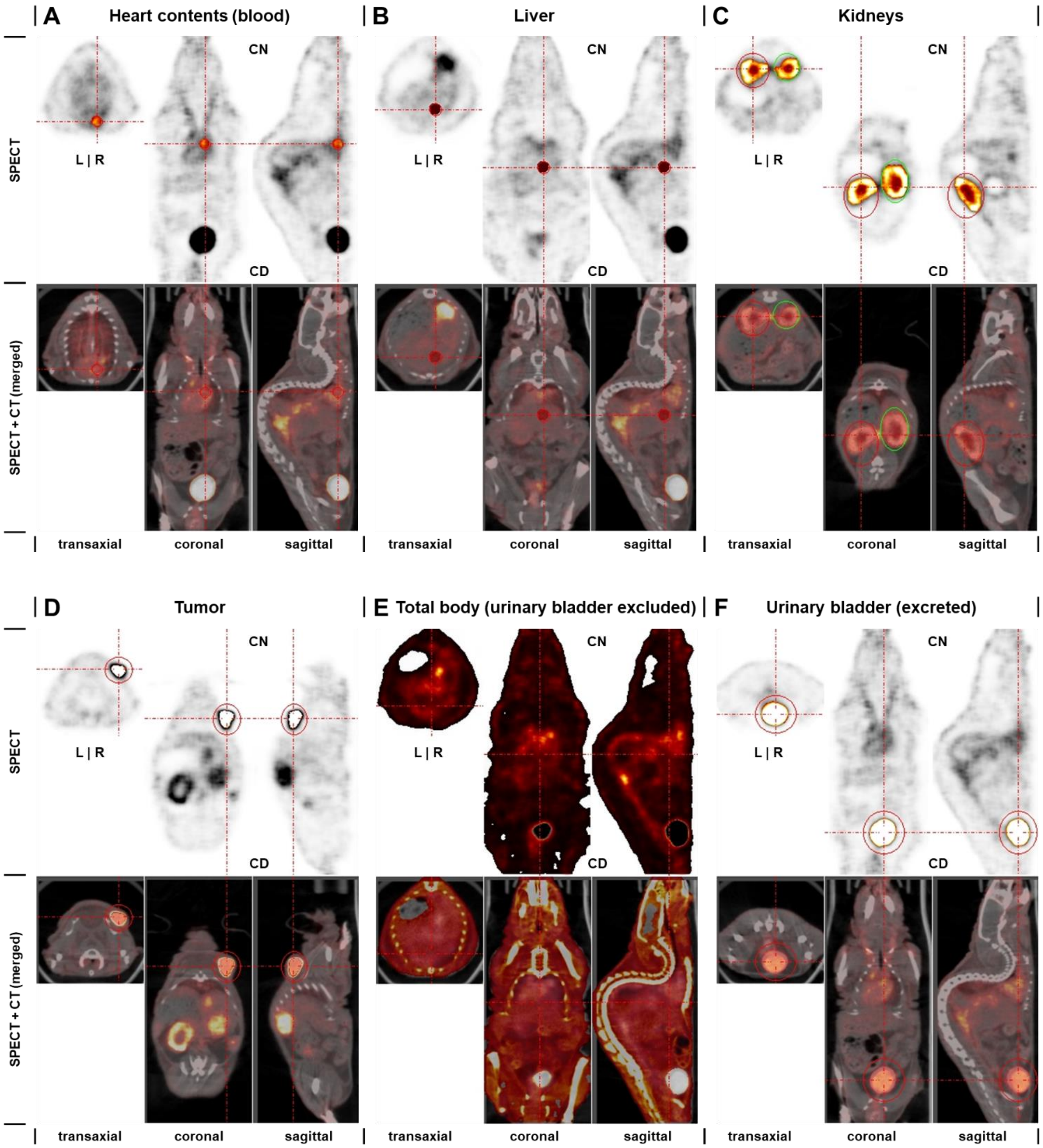

Figure S 8: Extraction of tissue-specific activity concentrations from SPECT/CT images of MPC allograft mice; positions of masks (red circles) and ROIs (yellow/white-highlighted regions) exemplified in orthogonal image slices of a SPECT/CT scan recorded 5 h after treatment with [^{67}Cu]Cu-NODAGA-cLAB4-TATE; intersections of red crosshairs indicate the positions of tissue-specific masks and ROIs; (L) left; (R) right, (CN) cranial, (CD) caudal

VIII. Pharmacokinetics of lutetium-177- and copper-67-labeled TATE variants in MPC allograft mice

Tissue-specific pharmacokinetic profiles in mouse organs and MPC tumors

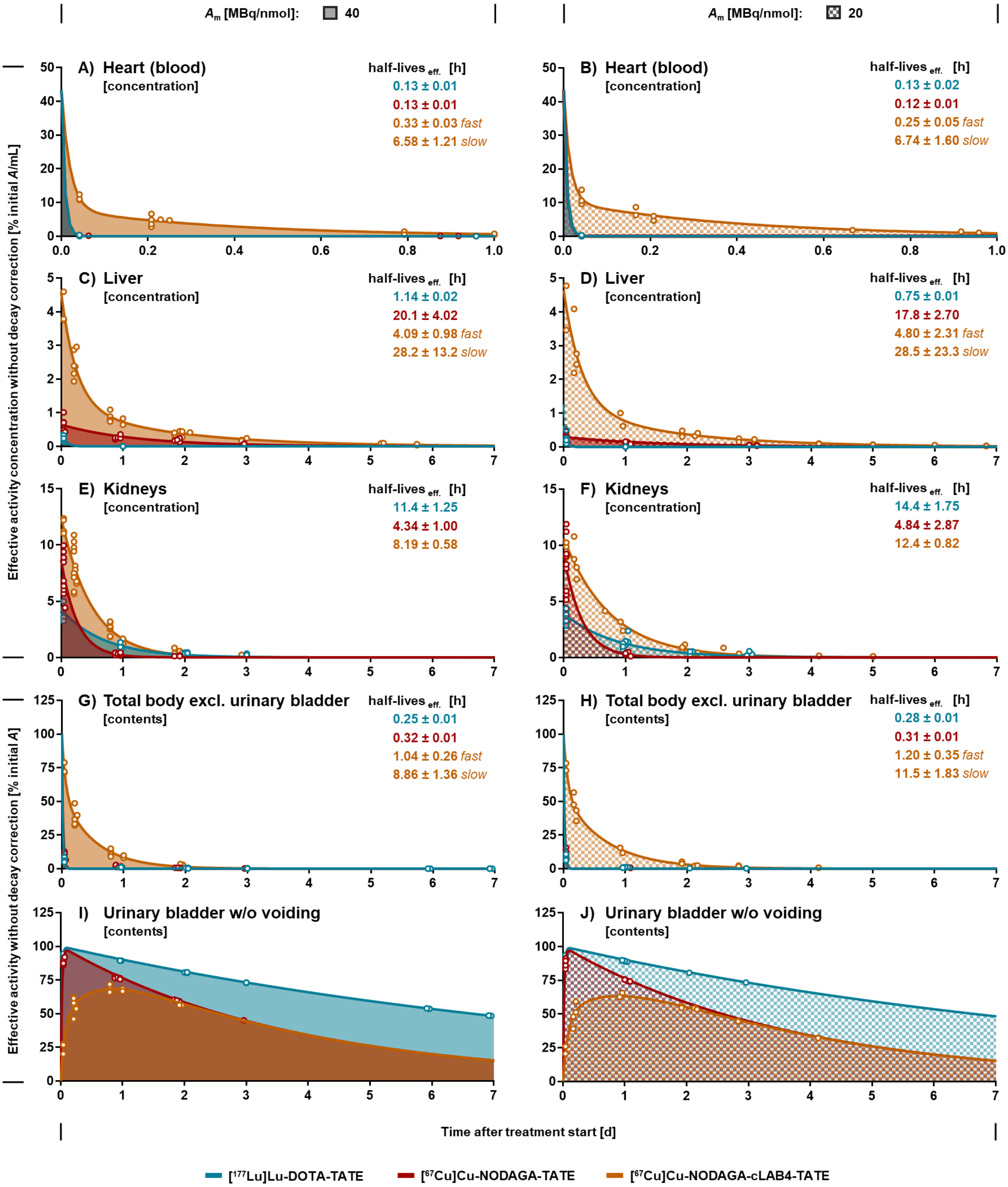

Figure S 9: Pharmacokinetic organ and excretion profiles of lutetium-177- and copper-67-labeled TATE variants in mice; (A–F) Organ-specific time courses of activity concentrations; (G–J) Time courses of excreted activity; animals received an initial activity dose of 50 MBq corresponding to molar amounts of 1.25 nmol (A_m = 40 MBq/nmol) or 2.5 nmol (A_m = 20 MBq/nmol); data presented normalized to initially injected activity dose and without decay correction

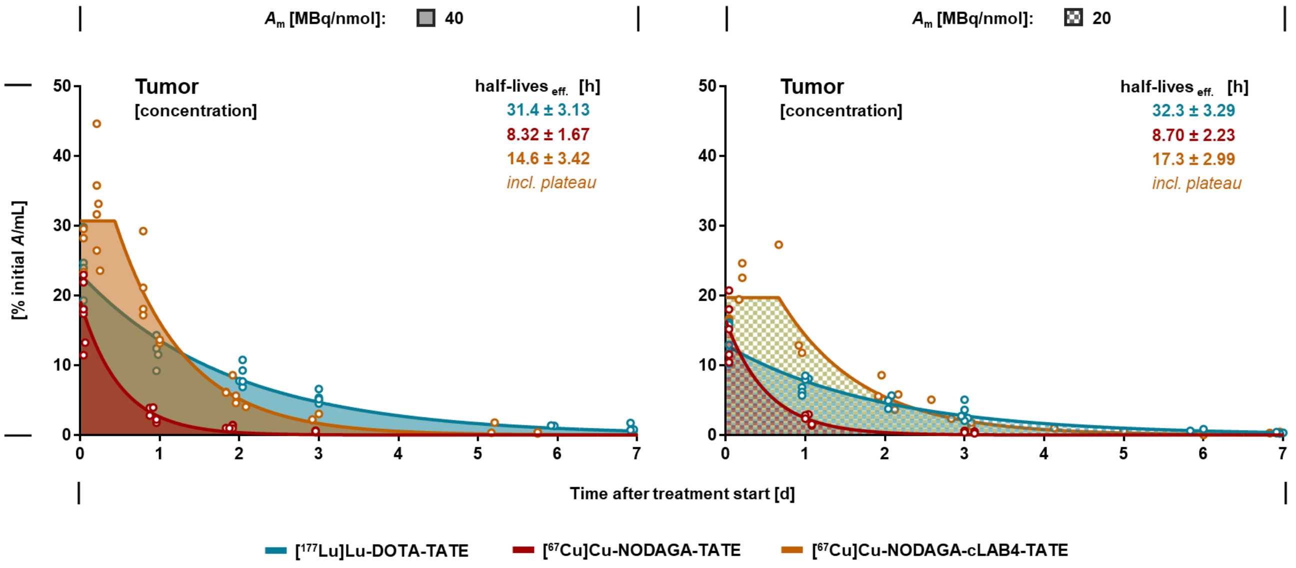

Figure S 10: Pharmacokinetic profiles of lutetium-177- and copper-67-labeled TATE variants in subcutaneous MPC tumor allografts in mice; animals received an initial activity dose of 50 MBq corresponding to molar amounts of 1.25 nmol (A_m = 40 MBq/nmol) or 2.5 nmol (A_m = 20 MBq/nmol); data presented normalized to initially injected activity dose and without decay correction

Table S 3: Organ pharmacokinetics of lutetium-177- and copper-67-labeled TATE variants (A_m = 40 MBq/nmol) in MPC allograft mice (decay-corrected)

**Organ**		**1 h**	**5 h**	**3 d**	**7 d**
**[177Lu]Lu-DOTA-TATE**					
Heart	[% ID/mL]	0.21 ± 0.18	0	0	0
Liver	[% ID/mL]	0.24 ± 0.04	0	0	0
Kidneys	[% ID/mL]	4.05 ± 0.01	3.23 ± 0.04	0.08 ± 0.01	0
Tumor	[% ID/mL]	22.7 ± 1.84	21.3 ± 1.72	6.79 ± 0.55	1.33 ± 0.11
Total body (excl. urinary bladder)	[% ID]	6.32 ± 0.61	0	0	0
**[67Cu]Cu-NODAGA-TATE**					
Heart	[% ID/mL]	0.35 ± 0.13	0	0	0
Liver	[% ID/mL]	0.63 ± 0.01	0.57 ± 0.01	0.15 ± 0.04	0.03 ± 0.02
Kidneys	[% ID/mL]	6.69 ± 0.46	3.87 ± 0.26	0	0
Tumor	[% ID/mL]	17.7 ± 1.83	13.5 ± 1.40	0.14 ± 0.01	0
Total body (excl. urinary bladder)	[% ID]	12.2 ± 0.94	0.01 ± 0.01	0	0
**[67Cu]Cu-NODAGA-cLAB4-TATE**					
Heart	[% ID/mL]	10.4 ± 0.75	4.97 ± 0.57	0.01 ± 0.01	0
Liver	[% ID/mL]	4.10 ± 0.01	2.64 ± 0.03	0.46 ± 0.07	0.23 ± 0.07
Kidneys	[% ID/mL]	11.4 ± 0.94	8.54 ± 0.71	0.07 ± 0.01	0
Tumor	[% ID/mL]	27.3 ± 1.91	34.5 ± 3.18	4.62 ± 0.33	0.23 ± 0.02
Total body (excl. urinary bladder)	[% ID]	73.3 ± 1.78	41.1 ± 2.69	0.44 ± 0.03	0

(% ID) decay-corrected proportion of initial activity dose; data presented as means ± standard error

Subcutaneous MPC tumor allografts are characterized by a rapid release of SSTR2 radioligands.

Subcutaneous MPC tumor allografts are characterized by a rapid release of SSTR2 radioligands. This is apparent from comparing the pharmacokinetic profiles of the clinical reference ligand **[^{177}Lu]Lu-DOTA-TATE** in MPC tumor allografts and other neuroendocrine tumor models in mice. MPC tumors exhibit higher initial uptake but also faster release of **[^{177}Lu]Lu-DOTA-TATE** (94 % within seven days) compared to other commonly used neuroendocrine tumor models with a natural SSTR2 biology, as reported for example for NCI-H69 tumor xenografts (68–85 % within seven days) and GOT1 tumor xenografts (47 % within seven days) [51-53, 85]. On the other hand, uptake and retention of **[^{177}Lu]Lu-DOTA-TATE** in MPC allografts are largely similar to the pharmacokinetic profiles reported for genetically modified subcutaneous HEK293-SSTR2+ tumor xenografts, especially in terms of a rapid radioligand release (95 % within seven days) [59]. In HEK293-SSTR2+ tumor xenografts, a comparably fast release has also been reported for **[^{64}Cu]Cu-DOTA-TATE** (86 % within seven days). This comparison indicates that pharmacokinetic profiles of SSTR2 radioligands are tumor model-specific, with considerable implications for treatment efficacy and thus prediction of their clinical performance.

IX. Projected human pharmacokinetics and dose estimations for treatments with lutetium-177- and copper-67-labeled TATE variants

Projected time-activity curves in humans extrapolated from pharmacokinetic profiles in mice

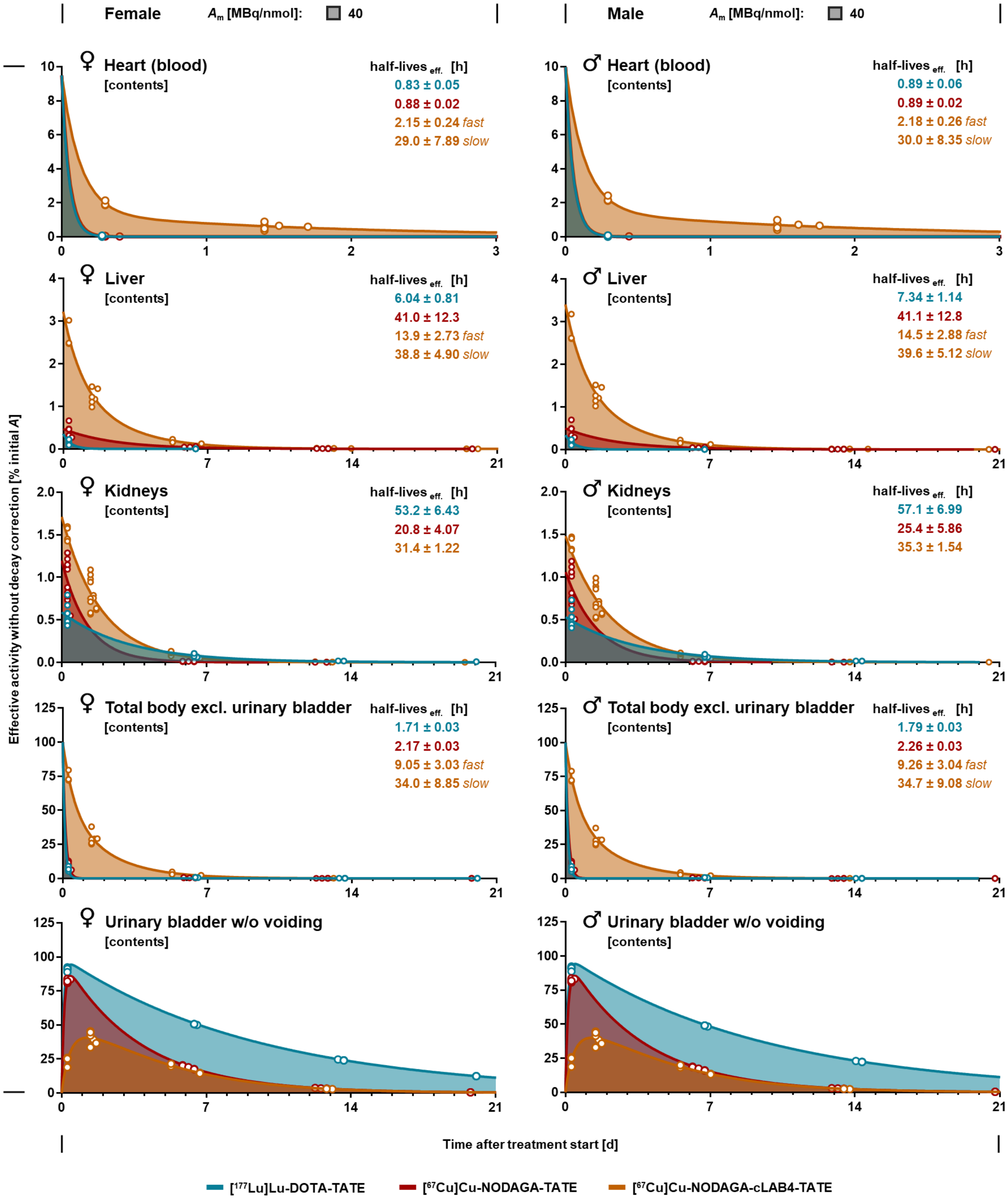

Figure S 11: Projected pharmacokinetics of lutetium-177- and copper-67-labeled TATE variants in female and male humans extrapolated from tissue-specific effective activity profiles in female MPC tumor allograft mice; animals received an initial activity dose of 50 MBq corresponding to a molar amount of 1.25 nmol (A_m = 40 MBq/nmol); data presented normalized to initially injected activity dose without decay correction

Projected human organ doses for radioligand treatments at a molar activity of 40 MBq/nmol

Table S 4: Projected female and male human organ doses (mSv/MBq) calculated from extrapolated radioligand pharmacokinetics

**Organ**	**[177Lu]Lu–DOTA–TATE** [a]		**[67Cu]Cu–NODAGA–TATE** [a]		**[67Cu]Cu–NODAGA–cLAB4–TATE** [a]	
	female	male	female	male	female	male
Adrenals	4.17E–03	4.25E–03	8.19E–03	8.12E–03	6.55E–02	5.73E–02
Brain	2.86E–03	2.50E–03	4.22E–03	3.68E–03	5.30E–02	4.43E–02
Breasts	2.85E–03	–	4.26E–03	–	5.16E–02	–
Esophagus	3.08E–03	2.70E–03	5.30E–03	4.64E–03	5.74E–02	4.98E–02
Eyes	2.86E–03	2.50E–03	4.23E–03	3.68E–03	5.30E–02	4.43E–02
Gallbladder	3.60E–03	2.81E–03	7.20E–03	5.86E–03	6.39E–02	5.41E–02
Left colon	3.65E–03	3.01E–03	6.84E–03	5.41E–03	6.22E–02	5.13E–02
Small intestine	3.86E–03	3.33E–03	7.52E–03	6.40E–03	6.04E–02	5.18E–02
Stomach	3.18E–03	2.76E–03	5.49E–03	4.78E–03	5.99E–02	5.07E–02
Right colon	3.34E–03	2.97E–03	6.02E–03	5.51E–03	6.07E–02	5.14E–02
Rectum	8.08E–03	4.91E–03	2.02E–02	1.10E–02	6.78E–02	5.34E–02
Heart wall	1.77E–02	1.37E–02	2.07E–02	1.67E–02	1.65E–01	1.37E–01
Kidneys	1.40E–01	1.20E–01	1.26E–01	1.04E–01	2.68E–01	2.33E–01
Liver	2.44E–03	2.17E–03	2.20E–02	1.80E–02	1.02E–01	8.63E–02
Lungs	3.04E–03	2.63E–03	5.11E–03	4.38E–03	5.85E–02	4.83E–02
Ovaries	5.22E–03	–	1.16E–02	–	6.35E–02	–
Pancreas	3.34E–03	2.85E–03	6.40E–03	5.18E–03	6.32E–02	5.28E–02
Prostate	–	5.85E–03	–	1.38E–02	–	5.45E–02
Salivary glands	2.87E–03	2.55E–03	4.29E–03	3.90E–03	5.36E–02	4.67E–02
Red marrow	2.66E–03	2.38E–03	5.01E–03	4.49E–03	4.58E–02	3.92E–02
Osteogenic cells	2.99E–03	3.34E–03	5.89E–03	6.19E–03	5.67E–02	5.81E–02
Spleen	3.64E–03	3.00E–03	6.36E–03	5.13E–03	6.05E–02	5.00E–02
Testes	–	3.20E–03	–	5.87E–03	–	4.63E–02
Thymus	3.07E–03	2.70E–03	5.06E–03	4.47E–03	5.90E–02	4.98E–02
Thyroid	2.90E–03	2.57E–03	4.42E–03	4.00E–03	5.42E–02	4.72E–02
Urinary bladder	6.62E–01	5.15E–01	6.65E–01	5.17E–01	4.49E–01	3.50E–01
Uterus	8.33E–03	–	2.09E–02	–	6.83E–02	–
Total body	7.62E–03	6.39E–03	1.02E–02	8.37E–03	6.23E–02	5.20E–02
**Effective dose**	**3.07E–02**	**2.39E–02**	**3.38E–02**	**2.60E–02**	**7.18E–02**	**5.40E–02**

[a] estimated for human females (60 kg) and males (73 kg) from quantitative analysis of SPECT images after treatment of female NMRI-nude mice with 50 MBq of the radioligand at a molar activity of 40 MBq/nmol; kinetic data input: heart contents, kidneys, liver, urinary bladder contents, total body remainder; data presented as means in mSv/MBq

X. Treatment effects of lutetium-177- and copper-67-labeled TATE variants in MPC allograft mice

Growth-reducing effects on subcutaneous MPC tumor allografts

Treatments with **[^{67}Cu]-NODAGA-TATE**, **[^{67}Cu]Cu-NODAGA-cLAB4-TATE**, and **[^{177}Lu]Lu-DOTA-TATE** were associated with temporary inhibition of tumor growth resulting in treatment-specific improvements in survival (Figure S 12). Radioligands administered at a molar activity of 40 MBq/nmol provided the strongest growth-inhibiting effects including an initial reduction in tumor volume for up to 10 days in response to **[^{67}Cu]Cu-NODAGA-cLAB4-TATE**, and **[^{177}Lu]Lu-DOTA-TATE**.

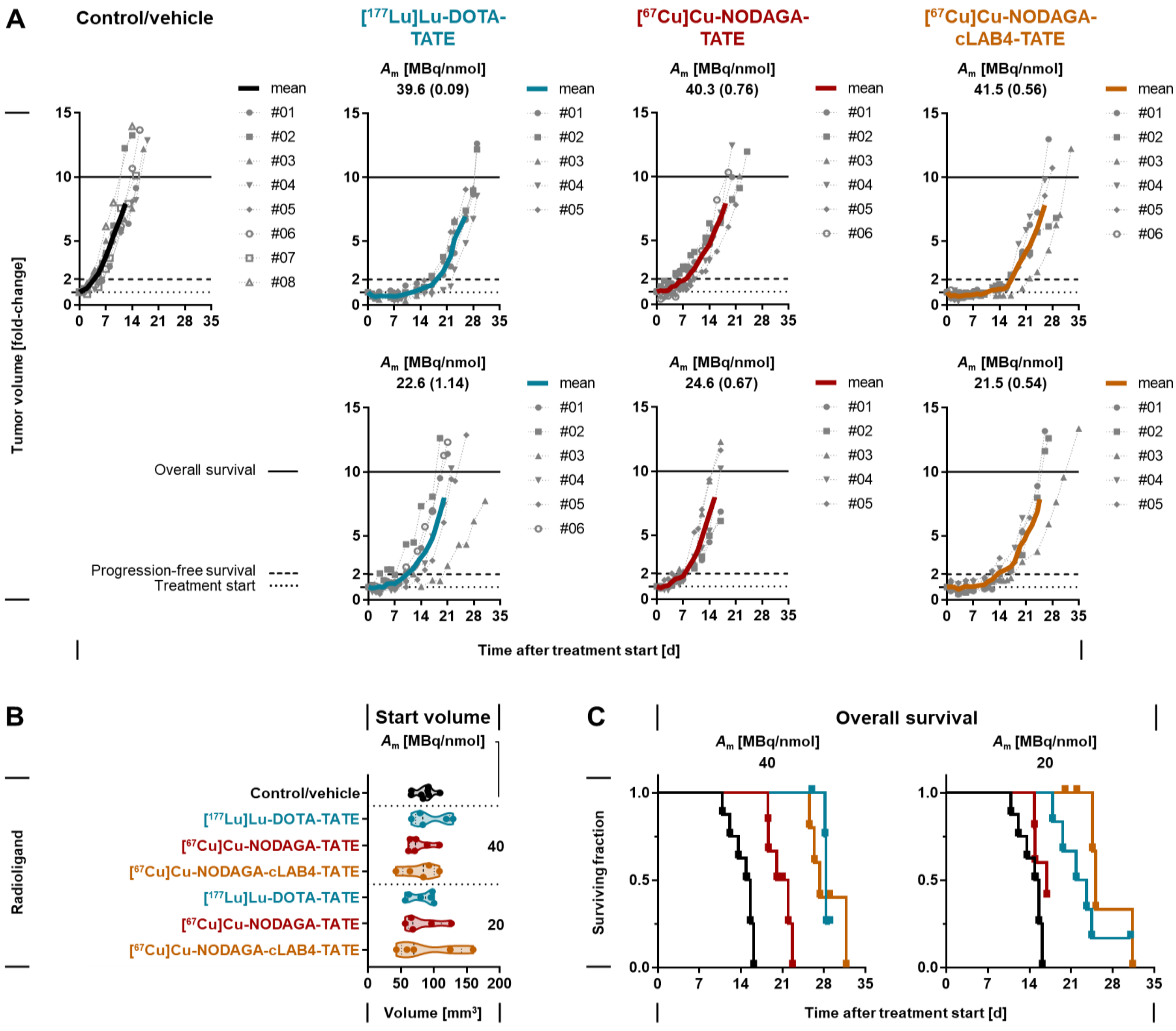

Figure S 12: Tumor growth and survival of MPC allograft mice treated with lutetium-177- and copper-67-labeled TATE variants; animals received an initial activity dose of 50 MBq corresponding to molar amounts of 1.25 nmol (A_m = 40 MBq/nmol) or 2.5 nmol (A_m = 20 MBq/nmol); (A) Fold-change in tumor volume after treatment; solid curves indicate the mean progression until ~8-fold change in tumor volume (> 60 % of animals available in each cohort); (B) Tumor start volumes in each cohort; (C) Overall survival of tumor-bearing mice is defined as < 10-fold change in tumor volume compared to treatment start; censored data points along horizontal lines indicate animals withdrawn from follow-up before reaching the overall survival criterion; (A_m) molar activity at treatment start; data presented as means with standard error

Absorbed doses in MPC tumors

The highest total absorbed doses in MPC tumors after single administration of 50 MBq **[67Cu]Cu-NODAGA-cLAB4-TATE** and **[177Lu]Lu-DOTA-TATE** in mice (40.2–41.1 Gy) are comparable to those in patients with SSTR2-positive NENs receiving one typical treatment cycle of 7.4 GBq **[177Lu]Lu-DOTA-TATE** [86, 87]. This allows, at least to some extent, a translational perspective on the responses of MPC tumors to the experimental treatments investigated herein. Nevertheless, treatment responses of subcutaneous MPC allograft models may differ from that of human PCC/PGL due to their higher growth rates and heterotopic location, as well as the immunodeficient phenotype of the NMRI-nude mouse strain.

Time courses in body weight of MPC allograft mice treated with lutetium-177- and copper-67-labeled TATE variants

Treatments of MPC tumor allograft mice with **[67Cu]-NODAGA-TATE** and **[67Cu]Cu-NODAGA-cLAB4-TATE** were well tolerated with maximum body weight losses in the 10 % range, similar to treatment with **[177Lu]Lu-DOTA-TATE** (Figure S 13). The largest decrease was observed in both control and treatment cohorts when tumor volumes exceeded 700 mm^3. This indicates that losses in body weight most likely resulted from the effects of tumor re-growth rather than treatment with the radioligand.

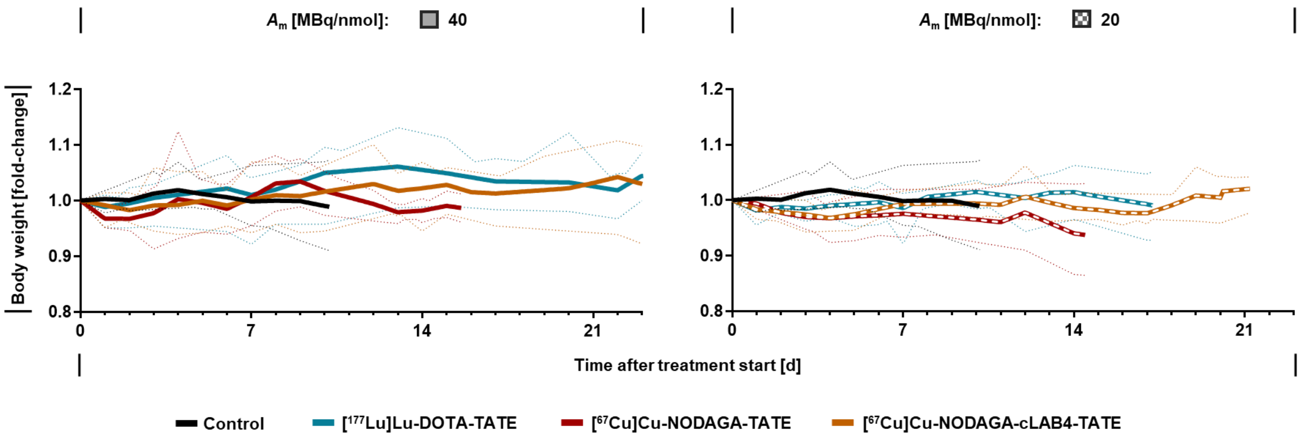

Figure S 13: Time courses in body weights of MPC allograft mice treated with lutetium-177- and copper-67-labeled TATE variants; animals received an initial activity dose of 50 MBq corresponding to molar amounts of 1.25 nmol (A_m = 40 MBq/nmol) or 2.5 nmol (A_m = 20 MBq/nmol); fold-changes in body weight relative to treatment start presented as cohort means (bold lines) with lower and upper limits (dotted lines)

All references within the supporting information are included in the main document.
